# “We are looking at the future right now”: community acceptability of a home-based viral load test device in the context of HIV cure-related research with analytical treatment interruptions in the United States

**Published:** 2022-03-29

**Authors:** Karine Dubé, John Kanazawa, Christopher Roebuck, Steven Johnson, William B. Carter, Lynda Dee, Beth Peterson, Kenneth M. Lynn, Linden Lalley-Chareczko, Emily Hiserodt, Sukyung Kim, Daniel Rosenbloom, Brad R. Evans, Melanie Anderson, Daria J. Hazuda, Lisa Shipley, Kevin Bateman, Bonnie J. Howell, Karam Mounzer, Pablo Tebas, Luis J. Montaner

**Affiliations:** 1 UNC Gillings School of Global Public Health, Chapel Hill, NC, USA; 2 Department of Science and Technology Studies, Cornell University, Ithaca, NY, USA; 3 Martin Delaney BEAT-HIV Collaboratory Community Advisory Board (CAB), Philadelphia, PA, USA; 4 AIDS Treatment Activists Coalition (ATAC), Nationwide, USA; 5 AIDS Action Baltimore, Baltimore, MD, USA; 6 Delaney AIDS Research Enterprise (DARE) CAB, San Francisco, CA, USA; 7 Wistar Institute and Martin Delaney BEAT-HIV Collaboratory, Philadelphia, PA, USA; 8 Hospital of the University of Pennsylvania, Philadelphia, PA, USA; 9 Philadelphia FIGHT Community Health Centers, Philadelphia, PA; 10 Merck & Co, Inc., Kenilworth, NJ, USA

**Keywords:** Home-based viral load, acceptability, HIV cure research, analytical treatment interruptions, people with HIV

## Abstract

People with HIV (PWH) and community members have advocated for the development of a home-based viral load test device that could make analytical treatment interruptions (ATIs) less burdensome.

We assessed community acceptability of a novel home-based viral load test device.

In 2021, we conducted 15 interviews and 3 virtual focus groups with PWH involved in HIV cure research. We used conventional thematic analysis to analyze the data.

PWH viewed the home-based viral load test device as a critical adjunct in ongoing HIV cure trials with ATIs. The ability to test for viral load at home on demand would alleviate anxiety around being off ART. Participants drew parallels with glucometers used for diabetes. A preference was expressed for the home-based test to clearly indicate whether one was detectable or undetectable for HIV to mitigate risk of HIV transmission to partners. Perceived advantages of the device included convenience, sense of control, and no puncturing of veins. Perceived concerns were possible physical marks, user errors and navigating the logistics of mailing samples to a laboratory and receiving test results. Participants expressed mixed effects on stigma, such as helping normalize HIV, but increased potential for inadvertent disclosure of HIV status or ATI participation. Increasing pluripotency of the device beyond viral load testing (e.g., CD4+ count test) would increase its utility. Participants suggested pairing the device with telemedicine and mobile health technologies.

If proven effective, the home-based viral load test device will become a critical adjunct in HIV cure research and HIV care.

## Introduction

The number of clinical trials aimed at finding a cure for HIV – i.e., the sustained virologic suppression in the absence of antiretroviral treatment (ART) – is rapidly growing.^1^ In most of these investigations, people with HIV (PWH) interrupt ART to evaluate the effect of investigational interventions.^2^ This pausing of ART is called analytical treatment interruptions (ATIs) and the need of a precise determination of the “time to viral rebound” can result in as many as two viral load test visits each week, resulting in high burden and inconvenience for trial participants.^3^ The duration and level of viral rebound can help determine when participants should restart ART.^4^ In some trials, viral replication is needed to induce immune responses to demonstrate the effectiveness of experimental HIV cure-related interventions.^2^ While research is being done to identify biomarkers that could predict the likelihood of this viral recrudescence,^5–7^ there is still no way to accurately predict when virus will return in the blood;^8^ therefore, frequent viral load test visits is warranted despite the inconvenience.

A home viral load collection device is currently under development by Merck & Co, Inc., Kenilworth, NJ, USA and Tasso, Inc.^9^ in the research setting. For the current device prototype, blood samples can be collected at home, but testing must occur in a laboratory. This home blood collection device could facilitate self-collection of plasma samples during ATIs. Community advocates and PWH have been advocating for the creation of such a home-based viral load.^2,3^ The ability to collect samples for viral load testing from home on demand and in a timely manner could reduce ATI trial burden, and help participants determine when they become viremic in the context of ATI trials. This critical knowledge could help reduce the risk of secondary unintended HIV transmission events to sexual partners during ATIs.^10–13^ Further, the device uses capillary blood draws and avoids the need for venipuncture. The device also decreases the collection volume, is simple to use and relatively painless.

The recent COVID-19 pandemic has precipitated renewed interest towards home-testing technologies to decrease the frequency of clinic visits to reduce the risk of SARS-Cov-2 exposure and transmission.^14^ Self-specimen collection methods have also been successfully used in previous HIV^15–20^ and sexually transmitted infections (STIs)^21–30^ research. Recent years have also witnessed the development of home-based HIV pre-exposure prophylaxis (PrEP) and support systems.^31^ Self-testing has made in-roads into other infectious diseases – such as tuberculosis^32^ and chronic, non-communicable diseases such as hypertension and cardiovascular health.^33,34^ Blood glucose meters and self-monitoring of diabetes have been in existence since the 1970s.^35^

To understand the acceptability of the current version of a novel home-based viral load collection device among community members involved in HIV curerelated research, we implemented a qualitative interview and focus group study. Our study corresponds to supportive behavioral and social sciences research (BSSR) aimed at strengthening the design and outcomes of biomedically-focused clinical trials.^36^ Formative research can inform whether proposed interventions would be acceptable to communities of interest and meet their needs. This research also fits within the U.S. Food and Drug Administration (FDA) framework for patient-focused product development.^37^ Understanding patient preferences can help developers refine device features. We hope findings will help inform implementation of the home-based viral load test device in the context of ATI trials and regular HIV care.

## Methods

### Study setting and participants

We used a purposive sampling technique to select potential participants for this qualitative study. Participants for the 15 community in-depth interviews and three virtual focus groups were recruited from community-based organizations (CBOs) and community advisory boards (CABs) in the Philadelphia area affiliated with the BEAT-HIV Collaboratory (beat-hiv.org), such as Philadelphia FIGHT Community Health Centers (FIGHT – fight.org). We also recruited participants affiliated with the Martin Delaney Community Advisory Boards (CABs) who regularly advise biomedical researchers on HIV cure research. To participate in the study, participants met the following eligibility criteria: (1) PWH, (2) affiliated with the BEAT-HIV Collaboratory, Philadelphia FIGHT or other community advisory group advising on HIV cure research in the United States, (3) ≥18 years old, and (4) willing to provide informed consent and share their opinion.

### Participant recruitment

We used an Institutional Review Board (IRB)-approved flyer to recruit potential interview and virtual focus group participants. Interested participants contacted the study coordinator (J.K.) by email or by phone to be considered for an interview or focus group. Participants completed a written informed consent form and a standard demographic sheet, before proceeding to the interview or focus group. Focus group participants were also briefed on additional data security measures, such as conducting the focus group from a private location where they could not be overheard or using a pseudonym.

### Data collection

**Figure 1.**
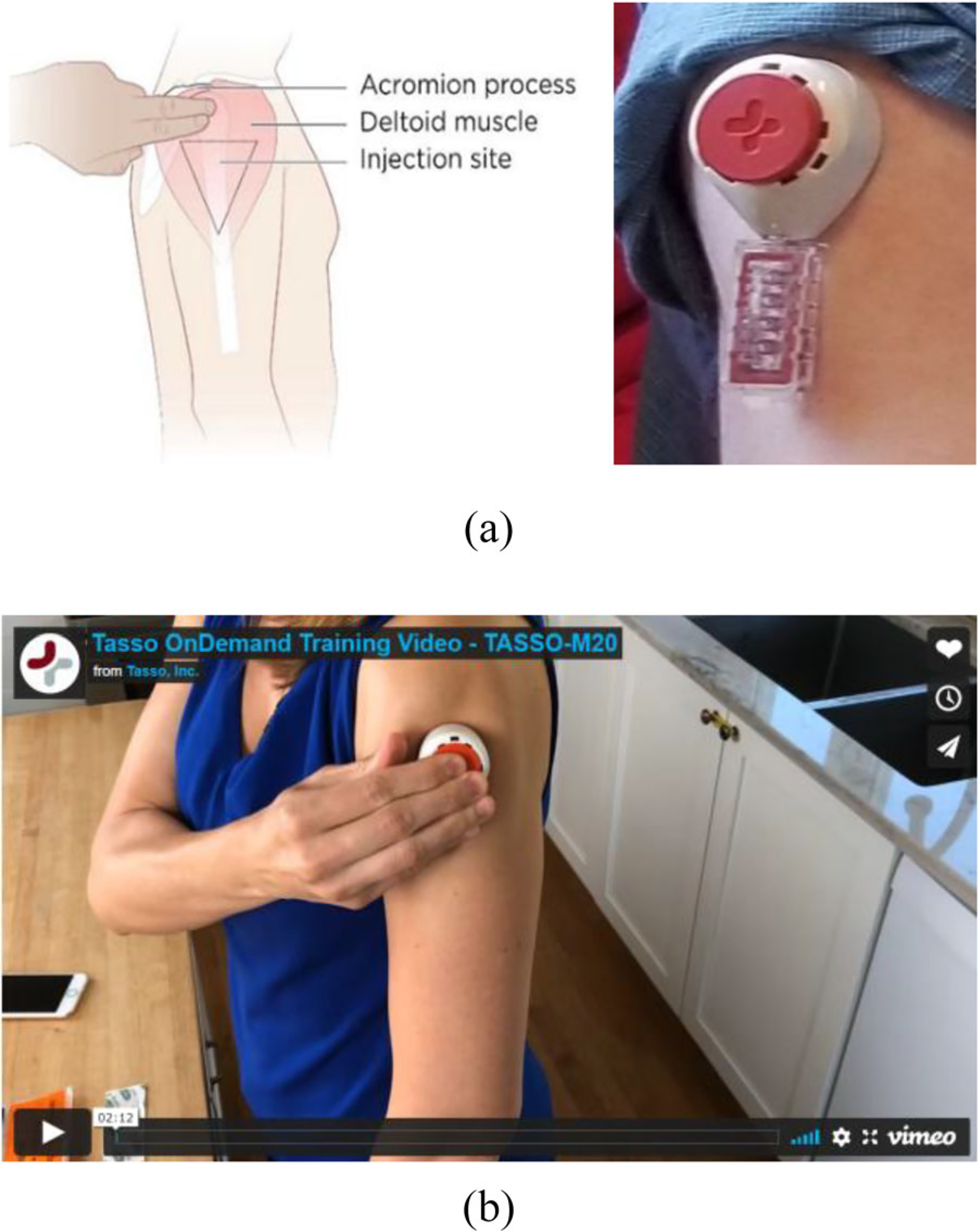
a. Experimental home-based viral load test device under development by Tasso and Merck & Co, Inc., Kenilworth, NJ, USA. b. Tasso home-based viral load test device demonstration video.

We developed the interview guide and focus group question route (Table 1) in close collaboration with members of the BEAT-HIV CAB and BEAT-HIV Social Sciences Working Group. In addition, a community member (L.D.) affiliated with the AIDS Treatment Activists Coalition (ATAC) reviewed the study instruments. Between May – June 2021, a research team member (J.K.) conducted all interviews in English, using a Health Insurance Portability and Accountability Act (HIPAA)-compliant virtual conferencing platform. Interviews took between 20–45 minutes. Similarly, research team members (S.J., J.K., C.R. and K.D.) faciliated virtual focus groups using a HIPAA-compliant platform, and these focus groups took place in May and June 2021 and lasted 90–120 minutes. We used IRB-approved PowerPoint slides to guide virtual focus group conversations. All interview and focus group participants received a copy of the home-based viral load test collection device demonstration video (Figure 1b), which was also IRB-approved. The video provided a description of the home-based viral load test device and kit contents. The device kit contained: instructions for use, the device itself (Tasso M-50), alcohol wipe, band-aid, and return pouch and box. Interview and focus group participants each received $50 for their participation.

### Data analysis

Interviews and focus groups were professionally transcribed. One research team member (J.K.) reviewed all transcripts for accuracy and completeness against the audio recordings. Due to the exploratory nature of the research topic, we used conventional content analysis involving inductive reasoning to analyze the qualitative data.^38^ Our goal was to reduce the data to inform acceptability and implementation of the home-based viral load test device in the context of HIV cure research involving ATIs.

A senior research team member (K.D.) compiled all de-identified text responses into one master document for manual coding. To realize the potential of the rich dataset, we analyzed the data by question blocks (i.e., question by question) to view the range of responses received. After careful review of participant responses, we extracted salient quotes, and ascribed codes or themes. Two research team members (K.D. and J.K.) double-coded the data, and organized text units into emergent themes. The codebook was inductive, containing code names and examples for each code. We expanded and collapsed codes as needed during data review. We resolved coding discrepancies by consensus during virtual meetings. Once the main codes were identified, we summarized key patterns and prepared narrative summaries. Findings were reviewed with the BEAT-HIV CAB and Social Sciences Working Group to identify salient themes from a community perspective.

### Ethics statement

This study was approved by the University of North Carolina at Chapel Hill (UNC-CH) IRB (study #20–2957).

## Results

### Interview and focus group participants

We interviewed 2 cisgender women and 13 cisgender men, of whom 8 were Black/African American, 5 were White/Caucasian, 1 was Hispanic/Latino and 1 identified with more than one race. Interview participants were between 33–64 years old (mean age: 52 years) (Table 2a). Two interview participants had previously participated in an HIV cure study involving an ATI. Virtual focus groups compromised of 5 cisgender women, 10 cisgender men, 1 transgender woman and 1 intersex participant. Of these, 10 were Black/African American, 2 were White/Caucasian, 1 was Hispanic/Latino and 4 identified with more than one race. Focus group participants were between 29 and 62 years old (mean age: 52 years) (Table 2b). Participant demographics are representative of PWH in the Philadelphia area.^39^

### Background context

#### Perceptions of analytical treatment interruptions in HIV cure research.

Most participants had prior general knowledge or awareness of HIV cure research occurring in the Philadelphia area and across the United States. One participant described reasons behind pursuing a cure, such as the high costs and long-term side effects of lifelong HIV treatment.

There is a need for cure research because even though we have effective treatments available which allow us to live with HIV for what has become pretty much a normal lifespan and a healthy life. The difficulties are that the treatments are not accessible to everybody. They’re expensive and not everybody who’s living with HIV has access to treatment. And secondly, even for those of us who do, there are long-term side effects and it’s costly, whether that’s directly out of pocket or insurance, it still costs quite a bit. And then, there’s ongoing effects from living with HIV and the medication. – CI-11

We asked participants to describe their views on ATIs in the context of HIV cure trials. Several participants were in favor of ATIs and expressed this would be the only way to test the efficacy of experimental interventions.

At this point in time, it’s the only way that we can find out whether the intervention, whatever it is we’re trying to assess, it’s the only way we can find out whether it works or not or how well it works. So, the treatment interruptions are needed. Hopefully, at some point, we’ll figure out other ways to test the efficacy of these cure strategies without a treatment interruption, but right now, we don’t have that luxury. So they’re necessary, somebody has to do it. – CI-11

I mean, I think you’re going to have to do it. There’s no way not to. How do you define if it’s working or not? You’re not going to willingly infect people and then try and cure them. You got to have people that are going to be off of medication in order to see if this is actually going to cure it, short-term, long-term. I mean, I guess, how do you determine what the cure is short-term, long-term because what is it? There’s concern that if you can cure it or you can get rid of the disease, the virus, but is it really hiding somewhere in some cell somewhere and all of a sudden, 10 years later, it decides it’s going to come back? I guess is it really cured? How do you tell that? Something’s got to be done. – CI-06

Some participants expressed skepticism or concerns around ATIs, such as increase in viral load, decrease in CD4+ count, development of ART resistance, and effects on concomitant medications. One participant even expressed being terrified of the prospect of interrupting treatment.

Well, on that one, I’m a little skeptical. Because I was taught that you got to take your meds, you got to take your meds every day, you got to take your meds at around the same time every day so that they could work, so that the virus doesn’t multiply. So, if I stopped taking my meds, then I’m giving the virus as a chance to multiply. I was also taught that the virus in the reservoirs and that’s where it’s hiding out, even when we’re undetectable, and I’ve been undetectable for 17 years. So, I’m a little skeptical about that. – CI-05

Terrifying. Right, because what have we been told? You don’t stop. You always take every day. Don’t miss. God forbid, because you’re going to create a strain that’s not going to be able to be controlled. When I read that I was like, “Whoa.” I would have a million questions about how that was going to work if I was going to participate in that. – CI-06

Several participants recognized the need for safeguards during ATIs, such as close monitoring of their viral load, and clear criteria for restarting ART. Participants would also want to receive approval from their HIV care provider.

I will have to discuss that with my doctor. I would probably think about it, but I will really have to discuss it with my doctor or something to see if it would be advisable or not. I really would, because all the information that I’ve gathered from the HIV meds and the resistant period, and all of that is scary alone, so I continuously take my meds. So to stop it for clinical research, I would have to ask my doctor is what I’m saying. – FG-1, Participant #1

Two interview participants had previously participated in an ATI trial, and recounted their experience. One participant (CI-10) became anxious during his ATI around having a detectable viral load in his body.

When you go on an ATI, the first thing is, it’s a delight, I’m not on meds. I mean I don’t have to constantly think, “Do I have my meds, have I taken my meds, have I ordered my meds,” but contrasted with that is after that euphoria dies down after about a day it’s like, “When am I going to become detectable?” The fear of transmission and not just transmission because for me, I’m in a committed relationship and I understand safer sex … but the fear of what’s going to, can I possibly become resistant to my meds? What about the, I guess the term is the irritation to my system from having a rebound and having virus loads in my system. I think that there are good sides to it, I think there are very scary and dangerous sides to it, but I understand the need for them and I support them. – CI-10

Most participants reported being vigilant with taking ART, but most had also ever missed a dose of medications. A few participants went long periods without ART due to personal circumstances (not in the context of research), such as addiction, financial or insurance issues, unstable housing or travels that precluded taking ART.

So, I am in recovery, and so, years ago when I was using, I would just stop taking it. It just didn’t fit into my lifestyle. But that’s years ago. And just more recently, I don’t think I’ve really consciously missed a dose, but it could’ve happened. Perhaps some, but I don’t think too much, and maybe the two months that I was using, and just stopped taking it, it wasn’t intermittent, it was just like, one day I was using, and I stopped taking it, and then I went into rehab, and started, again, and just continued.– CI-19

In the past, when housing wasn’t so stable and I had to do a lot of moving around, there were times that yes I was missing my medication. But once I became stable in my home and didn’t have to worry about moving and so on, I was fine … I think I missed a dose. I’ve missed one dose or two doses this year so far. – CI-09

Participants recognized the need for ATIs in the context of HIV cure research, and these views were calibrated based on personal experiences with previous HIV treatment interruptions outside of the research. Participants also acknowledged the need for ATI safeguards, including careful viral load monitoring during ATIs.

#### Experience with viral load checking.

Participants described their current experience with viral load checking. Most were conditioned to go to the clinic or laboratory every 3 or 6 months for blood draws.

Like I said, that’s something that’s never going to change … It’s just something that I have to do. So I’m just in the mindset of get it done, get it over with. I’m good with that. – CI-09

Most participants described the inconvenience of scheduling blood draws, including time commitments and logistical hurdles. Several participants described their aversion to needles or anxieties around blood draws that required venipuncture, because they were a ‘hard stick’. Other participants had poor veins due to prior addiction.

I wish there was a way they could find a way of drawing blood without using the veins. Because they never find my vein. So I’ve had to go to have my viral load blood withdrawn, and they could not find a vein after four sticks. And they sent me to come back another day. And then scheduling for another day is a real inconvenience because probably that’s the only date that you had in the course of your work. – FG-2, Participant #4

Because I’m a hard stick, I always have anxieties around the blood draw. And I’m really particular about how many times they’re going to stick me. And I let people know that upfront because I’m not a pin cushion. So I always have anxieties, not about the fact that the results of my viral load is going to be unacceptable to myself because I’m undetectable. And I have been that way for years. It’s the hard stick that I am. So I have a lot of anxieties around the actual process of drawing my blood or trying to get my blood out of my body so I can get my numbers read with my viral load. – FG-2, Participant #1

So, what do you need to know about me is I’m a recovering heroin addict … So now, being a recovering heroin addict, my veins are destroyed. So, they always have to use a butterfly and go in my hand. In the beginning, there was still some left in my veins at the top. But, the over the years that’s … So, it’s always a issue for me to get blood drawn. So, when I get somebody that can do it without … Well, see, first of all, you only sticking me three times. After three times, I’ll do it my damn self. So, it’s always been an issue. – CI-05

Other participants expressed aversion to having blood drawn, and the social risks of being identified as someone living with HIV when regularly going to see a phlebotomist.

Blood has always been embarrassing, because I feel like everybody knows, the doctor knows, and you get up there, and the person taking the blood, they know what them tubes are for. They know. Somewhere, they know. And then the other part about that is, them places that we go for our treatment, some people live out in the community. They’re going to see us. If we go to that clinic all the time, they’re going to see us and they’re going to remember us, and that’s going to stick in their head. – CI-07

The COVID-19 pandemic has generated further fears of going to the clinic for blood draws.

During the pandemic, I didn’t want to do it. I was like, “I’d rather just stay away from center city and everything.” So, when the pandemic was really in the swing, it was weird because I mean, earlier this year or late last year, somebody, I think might’ve had COVID that came into the office and had every symptom and I’m like, “I don’t want to be here.” – CI-14

Participants had incorporated in-clinic blood draws into their regular routine but wished these did not require finding or puncturing veins. The COVID-19 pandemic also generated additional anxieties around going to the clinic. The presence of a home-based viral load test device could help alleviate the burden of going into the clinic.

### Perceptions of home-based viral load test device

#### Perceptions of the device.

We asked participants to provide their initial impressions of the home-based viral load test device. Most participants commented that it was easy, practical, and reasonable to use.

I mean, it’s a typical scientific device, perfectly easy, perfectly simple. You always wonder if it’s really that easy and that simple to do and there’s no pain. I mean, it doesn’t look like it’s that … Is it really that easy? – CI-06

Some participants commented that a home-based viral load test device would be necessity as part of an HIV cure trial involving an ATI. The ability to test for viral load at home would help alleviate anxiety around being off ART.

I think if you’re involved in a clinical study such as this [with ATI], I see where it would be necessary to check it at home instead of a clinic. – CI-02

I think this device will help for that anxiety people would get around coming off of their medication. – FG-2, Participant #1

In addition, the device was perceived to be timely, innovative, and progressive. Words like ‘mind-blowing’ and ‘revolutionary’ were used to describe it.

Oh my god, for me, that’s mind blowing. I don’t have get stuck in my hand. I don’t have to walk around with one vein sticking out in my hand looking purple. Yes, that would be great. So, that would be something that would be just totally revolutionary for me … I think that thing is just marvelous. For me, I think it’s genius. I don’t know what took them so long to figure it out. – CI-05

I believe we are looking at the future right now. I think this is the future of viral load tests. I think five years from now, everybody will be doing this. This looks like we’re looking at the future. – FG-2, Participant #5

Several participants expressed having a sense of efficacy around being able to use the device. One focus group participant cautioned letting HIV care providers know about their use of device.

Yeah, I felt like it was comfortable. It gave me some security in knowing that I can do this myself and that this isn’t a big deal. – CI-09

I think for the first time I would rather use it with my PCP [primary care provider] … So in discussing it with my provider and having an open dialogue about the device, you don’t think that their approval will be given? – FG-1, Participant #3

One participant with diabetes drew a parallel between the device that allows him to monitor his blood sugar and the home-based viral load test device. Home-based devices provide him a sense of control around the ability to self-monitor his health.

I loved it because it reminds me, I’m a diabetic, so I don’t like sticking my fingers to take my levels. I use what they call a freestyle device … It’s a small little needle on the end, just like the device there … And it’s doable. I like it because I already do that to monitor my diabetes with my freestyle monitor. So when I was watching it, I was like, oh, I basically do that already. The only difference is putting that in, waiting for five minutes for the blood to come, and taking it off and going through the process of pulling it off and putting it back in the protective bag and putting it back in the box and dating it and sending it off. That was easy. I love it. – FG-1, Participant #1

Two participants, however, discussed how home-based viral load testing may not appeal to everyone. For example, several PWH enjoy coming to the clinic and interacting with research staff during their blood draws.

I believe that it will not be for everyone. I believe that wholeheartedly. I think you’re going to have a great number of people that will definitely find it helpful, and would definitely love to use it, especially the ones that want to be in the trials … But then you’re going to have other ones who are just, they won’t comprehend it, and they enjoy the idea of going out, and sitting there, talking with that coordinator or that person drawing that blood. – CI-13

Several participants commented that a home-based viral load test would have been helpful in relation to the COVID-19 pandemic.

It would have been the perfect test because it stopped people from going out and exposing themselves to the disease. And you still getting your accurate results, so that would’ve been perfect. – CI-17

One participant would prefer if the home-based test could clearly indicate whether he was detectable or undetectable for HIV directly at home, instead of having to send blood through the mail. This would allow him to assess whether he can transmit HIV to sexual partners during an ATI.

I would like a home test, different than what we’re talking about, that just, it’s something that you do at home like a strip of paper and it just says, “Detectable,” “Undetectable.” I mean because even with this, the test I watched the video on, you still have to mail it home, mail it back to somebody, and wait for a result. It’s the waiting and the time period of not knowing whether you’re detectable or not is the real downside, so there you go. However, the goal should still be, let me be very clear, a home test. This ain’t what I, as a community person, considers a home test. – CI-10

Overall, the home-based viral load test device was well-received in the community. Participants described it was easy to use, necessary, timely, and innovative. Participants made comparison with similar tools used in other indications (e.g., diabetes). Not all participants perceived the same level of utility, however. A test that could let PWH know immediately whether they are detectable for HIV and able to transmit the virus would prove beneficial.

#### Perceived advantages of the device.

Participants viewed several advantages of the home-based viral load test device. The simple convenience of being able to test viral load at home emerged as the strongest theme, particularly in the context of ATI trials.

I just wanted to say that right now, I believe this is being used by people on ATI trials, which the convenient part of it is that they need to be having their T-cells and viral load taken a heck of a lot more than any of us. I think they need it done like every week. So this device right now is excellent specifically for ATI. – FG-02, Participant #2

Other major attributes of the device were the time and cost-saving aspects.

I think it’s great in that aspect, or in a time crunch, if you’re working too much and you don’t have time to always take off work. I think it’s great for that. If anything, it’s a time saver and it’s not consuming. It’s not going to be consuming in my life. – CI-09

It’s saving you a visit to the doctor. That is a big thing. Because sometimes I just need to go and get my blood drawn … They take my money every time. So, that would say, I mean, I think it would save me some money on doctor visits. So, I’m thrilled with this idea. I’m really thrilled with it, personally. – CI-05

Participants further valued the sense of control and independence that would come with self-monitoring one’s viral load as part of an ATI trial.

That means we would feel more in control of what’s going on, what’s being done to us. Give us back some control. – CI-07

So I’m looking at some of the strengths of the device, one of them, if you go for it, is that it’s very empowering. You’re taking charge of your life in your own hands. You’re making a decision, you are proactively involved in your healthcare. So I loved that bit of it. – FG-2, Participant #4

Participants appreciated the safety of the device and the fact that it did not require puncturing the veins. Several participants commented that this would prove life-changing for individuals with poor venous access.

I think that will be incredible, especially, for people like me who struggle to have … Have to go through that uncomfortable pain of being poked many times just to find their vein, so yes. I wouldn’t have to worry about whether they’re going to find my veins or not. I can just quickly do it by myself at home. And I don’t have to worry about dealing with anxiety, or just going through all that process. – CI-18

Finally, participants appreciated that the device was discrete and did not appear painful. For most participants, the benefits appeared to outweigh the concerns of using the device.

#### Perceived concerns about the device.

Participants articulated potential concerns about the device. Several participants were concerned about the possible side effects, such as bruising or physical marks that would be left by the device, and the healing process.

It looked like it would leave a permanent scar there. What are the risks or how long will it take before that heals back up or how often you have to do it? – FG-2, Participant #3

Participants mentioned concerns around removing the device from the arm (e.g., blood leakage). Other concerns related to the fear of using the device incorrectly, the potential for contamination, delay with blood coming out and device defect. Participants wanted to ensure high quality viral load readings. Some participants were concerned the mailing would alter the quality of the sample.

Fear that I might not do it the right way, in the way that is supposed to be done. The contamination, fear of contaminating the device. Pretty much what I said already about fear of whether it’s going to be contaminated, or whether I’m going to do it the right way or not. That’s the only thing that normally you are concerned about. But other than that, I don’t think I have any other reservations about it. – CI-18

Given it’s in … viral load testing or measuring RNA [ribonucleic acid], which is really not that stable. And I’m just concerned that you’re getting blood into some chamber. I’m assuming there’s some stabilizer in there to help it, but then once you put it in a box and you ship it, it’s subject to so many lengths of time to get from point A to point B. You have different weather conditions. It could be in a truck that goes up to 180 degrees. How is this going to degrade, and I would have to see the sensitivity. Without seeing the sensitivity in comparison, I could not feel comfortable because especially in a cure trial, when you want to know what is the absolute value, you can’t mess that up at all. If it’s wrong, then it has no value.

– FG-3, Participant #1

Several participants also discussed mailing issues, such as having packages get lost in the mail or sent to the wrong address (discuss further below). Others were worried about not having enough money to pay for postage. Participants were concerned around turnaround times for receiving test results.

My main concern would be the time that the blood samples get to the lab to read properly, that transition. How will it get there? Will it get there in a timely matter so that way it’ll be able to be ready and be drawn? That’s a major concern. Because with my doctor, she always told me don’t come into the office on a Friday because then the blood samples will sit and then they won’t be as strong as they need to be for if you go in on a Monday, where they can send it off to the lab immediately. So that’s my main concern is that timeframe in between. – FG-2, Participant #3

Participants highlighted potential economic concerns of using the device. For example, in the context of an ATI trial, the device may translate into fewer financial incentives for study participants. Others were worried the device would not be covered by insurance.

Here’s my honest answer: I would love to do that instead of going into the clinic, but put it like this, when I come into the clinic, like say I’m doing a trial, and I come into the clinic to get my blood drawn, that might be a $50 day, or a $60 day. Now, if I do it at home, is it going to change, or once you get it will it still be the same? Do you know what I mean? So that’s important. I’m trying to think of every aspect as you ask me. – CI-13

I don’t know if there’s a cost associated with your insurance. And for me, a concern would be, would insurance approve this? Because everyone doesn’t have the same insurance, or some insurance that you got to get pre-authorizations for such things. Even if your doctor thinks this is a good thing for you to have, you still got to deal with your insurance company. So having a conversation with insurance companies about making sure that people who have any kind of HMO [health maintenance organization] or private insurance, that it’s available to them and that they approve it. And how many people need to jump through so many flaming hoops just to get this. Because it’s a great idea. – FG-2, Participant #1

Additional worries included the fear of forgetting to use the device and concerns that the device could lead to further social isolation, particularly during the COVID-19 pandemic.

#### Facilitators and barriers to using device.

We asked participants to describe facilitators and barriers to using the device. Facilitators included good orientation for use (e.g., video), and device features such as small size and easy grip.

I really couldn’t see, even a person with arthritis, I couldn’t see them having a problem with this device because it has a nice easy grip. There’s not a whole lot of hand eye coordination. You stick it up to your shoulder and here you go. – CI-09

Additional facilitators included having indications that the device was used correctly, use of incentives, and a reminder system such as text messaging. One suggestion was made to employ telemedicine to facilitate counseling of ATI trial participants on device use and safe sex in case of detectable HIV.

Also, there’s typically some sort of an interview and with an ATI trial, discussion about safety for any sexual partners. So again, how would that be done? It could be done by telemedicine but I think how that is done matters because again, if I can do that from home as well, then having the device may be a good thing. – CI-11

Participants noted several potential barriers to use, including blood aversion, hesitancy towards performing self-medical procedures, or concurrent medical issues such as anemia.

I think that the problem is people who are afraid of blood and already are afraid of blood draws. That’s a psychological thing. There’s no way around that. There are people who are not going to want to self-administer any kind of prick. That’s the only thing. – CI-10

Other barriers included transportation issues for mailing the blood and language barriers for minority populations.

#### Considerations for viral load test sensitivity.

Participants were informed that the current level of quantification for most commercial assays is between 20 and 50 copies per milliliter (mL). We also mentioned that the Undetectable = Untransmittable (U = U) campaign uses approximately 200 copies per mL as the level at which people may be able to sexually transmit the virus.

Most participants wanted the home-based viral load test to be as sensitive as possible. Some mentioned that having the test be as good as a clinic test would increase acceptability of the device. Some PWH are also concerned with viral blips.

I mean they should continue to strive to make it more sensitive, if possible. I think that the more sensitive it is, the more people will buy into it. Again, I always get concerned when I see a number over 20 or 50. I don’t see it that much but I would be concerned so I think that, and I don’t know how much harder or cost-prohibitive it would be to do that. – CI-10

I think that the tests should run about the same as the medical providers’ tests and it should read about the same. If you’re going to test it, you should test it. It’s equal. – FG-1, Participant #1

Several participants mentioned that the U = U benchmark would be acceptable. They suggested providing education to ATI trial participants around the need to protect sexual partners, and consulting with HIV care providers and research teams around when to restart ART.

I think it would depend on when and why it’s being used. So if it’s being used specifically in an ATI study, then one of my questions as a participant would be, am I still undetectable? Or is my viral load over 200 and now I need to be concerned about the possibility of transmitting to somebody else, if I were to have sex? So I think for that purpose, 200 would be the level that would make a difference. – CI-11

Only one interview participant mentioned levels higher than 200 copies/mL as the acceptable sensitivity threshold. Overall, the viral load test sensitivity would affect acceptability of the device. Being able to self-assess for transmissibility of HIV would appear to be important in the context of ATI trials.

#### Frequency of testing.

Participants shared their thoughts on the frequency of home-based viral load testing in the context of ATI trials. Several participants suggested once weekly home-based viral load testing.

Excellent question, so in my study which was an earlier ATI study, blood draws were at the beginning, every two weeks. That’s way too much time, in my humble opinion. I think that definitely once you start your ATI it should be weekly, and then I think that they have to be at least weekly. I think that that is something that our participants and the study team should talk about and assess each participant’s comfort level. – CI-10

Other participants recommended bi-weekly, once-monthly or every 6-week testing. Participants discussed factors that would affect the frequency of testing, such as turnaround time for viral load test results, study intervention or trial duration, and individual worries around being viremic.

There’s no answer to that because too often [this] depends on the participant. I mean that’s why there’s got to be flexibility built in. There may be people who are comfortable saying every day. Again, it’s a balancing game because if somebody is so worried that they want their blood tested every day or every twice a day, are they the right person to be in a clinical trial where we’re asking them to go off their meds. – CI-10

Building flexibility into trial design around home-based viral load testing was important to several participants.

#### Possible effects on stigma and social risks.

We asked participants to describe possible effects of the device on HIV-related stigma. Several participants expressed that being able to self-test viral load at home would reduce both external and internet stigma by limiting the number of clinic visits, providing a sense of control, and helping normalize HIV as a chronic condition.

I think it would probably lessen the stigma. I think it would make it a little less condemning because, one, you don’t have to run off to all these doctor appointments. You don’t have to explain a lot of things. When you can take power into your own hands a little bit more, it makes a person feel a little bit more secure and a little stronger about what’s going on with their bodies, what’s going on with their lives. – CI-09

As for the stigma, and thinking about the whole community, it might help the community see that this is something that you could live with. It’s manageable, more manageable than they think. And you don’t have to be afraid of people who have HIV, because there are many people who are still afraid of us because we have HIV, but I think it would be good for the community. – CI-07

And when it comes to stigma, this is really great. We’re normalizing getting our viral load done. This is amazing, amazing, amazing. And we’re just telling others that it’s just by getting your sugar level done at home or whatever.

– FG-3, Participant #4

Two participants noted, however, that the device could potentially augment stigma due to increased bruising on the arms, or leading PWH to become less adherent to ART if they are able to self-monitor viral load.

Well, I did a study a week ago. I’m a little bruised, this tiny little bruise and I had some bruising and some people who thought I shot needles, I’m like, “No, I don’t shoot up needles.” But I got some … gave some plasma for an HIV study about maybe I’m going to say almost a month ago. And so doing that, that might be stigma. “Well, what the hell? Are you diabetic? Or is this shooting something into you or?” Yeah. So, those markings might … Yeah. People will say things. – CI-14

I have a flip side to that question which I don’t know if you’re getting to, because I think there is a possible detriment to this, whether it would lead people to being a little bit less compliant because they say, “Oh, I can miss a few doses because I have a home test and I can quickly find out if I am no longer undetectable.” I think that that’s an important thing that should be added into the pot of things to think about. – CI-10

Several participants appreciated the discreteness of the device, and suggested it be treated like HIV medications in terms of protecting one’s privacy. A recommendation was made to include a disclaimer on the package.

It’s the same thing as people are going to be on meds, they’re going to be taking pills. I’m sure if they’re not comfortable, they’ve got to hide their pills, then add, even just add a disclosure sentence saying, “This is a personal medical device, you may want to consider how you store and reveal it to other people living in your residence and in your cupboard.” – CI-10

Possible social risks of the device included inadvertent or forced disclosure, especially for individuals in shared living situations. Frequent mailings or visits to the post office may also lead to disclosure.

#### Additional potential uses of the device.

Participants provided suggestions for additional uses of the device besides viral load testing in the context of ATI trials or regular HIV care. Most participants identified great potential for additional applications and wanted the device to provide measurements beyond viral load, such as CD4+ count, HIV resistance testing, chemistries, cholesterol, and/or blood sugar levels.

During my ATIs, I did testing every two weeks and I was fine with that and not particularly freaked out over having a detectable viral load. I’m more concerned about keeping track of my CD4+ count. I’m confident that the medications can bring the viral load back down to undetectable, so I’m honestly not worried about that. I know that it takes time and effort for my body to return CD4+ levels, to rebuild the CD4+ levels. And so, I’m more concerned about keeping an eye on those. And I don’t know whether that would also be tested with this home device or whether it’s just viral load. I guess that would limit the appeal of the device to me personally, because I’m more interested in the CD4+ level. – CI-11

Right. So, it could be to give you … What do you call it? Instead of just the viral load and the other HIV-related readings, it could be used for other type of blood work readings. I get a whole workup with the every six months, so they go over lipid panels and whatever else I normally get. Cholesterol readings and all of the everything else. It’s probably like, two or three tubes of blood work, which is a lot. I’d be afraid of all that coming out of my arm, but that’s basically it. So, if you can find a way to get more readings out of that blood work, then it would be worthwhile for me to take it twice a year when I get my blood work taken. – CI-19

Some participants expressed interest in having their blood tested for sexually transmitted infections (STIs), such as hepatitis or syphilis.

For hepatitis, for STDs [sexually transmitted diseases], for everything that blood needs to be drawn for, I believe that this would help. – CI-09

Participants also described specific populations or groups who could benefit from the device. These included people with needle aversion or poor vein access, those with phlebotomy fatigue, older individuals or people with disabilities or transportation issues, homeless or unstably housed individuals and people living in rural areas. Being able to test for more than viral load would increase acceptability and versatility for the device.

### Additional logistical considerations

#### Considerations for mailing in samples.

Most participants were comfortable with mailing in their samples and found the approach convenient. Nonetheless, potential concerns were raised around speed of shipping, packages getting lost in the mail or misplaced, damaged, or opened. Participants were concerned that extreme temperatures could affect the quality of samples. One participated noted cultural beliefs that could preclude shipping bodily specimens in some communities. Another participant raised concerns around sending deoxyribonucleic acid (DNA) in the mail.

Many participants preferred using a private company (such as FedEx or UPS) for mailing in samples. They viewed these companies as more reliable than the United States Postal Service and appreciated the ability of having specimens tracked and reliably delivered. Concerns were raised about the reliability of the regular postal service in recent years.

Now, that [private company] would be the ideal thing … I know they have boxes that you can drop them in, but sometimes you have to take it to a certain place … So, it wouldn’t be any problem for me. But, I think that sending it Federal Express or something like that would be perfect because that way you know it’s going to get where it’s got to go. – CI-05

The post office has lost a lot of credibility during this presidential elections about ballots disappearing and all that. And the conspiracy theories are still there. So how do we demystify, I mean, how do we turn down the conspiracy theories and convince our community that we can trust the post office? – FG-2, Participant #4

Additional considerations for mailing in samples included de-identifying packages to protect confidentiality, having no mention of HIV, and pre-stamping packages. Participants raised the issue of mailing in samples on the same day and providing several options to participants. One participant suggested including a mailing checklist in each test kit.

I like systems, so if there was maybe like, a card that would go in with that, that says, okay, you have the device in here, that you have your name or whatever numbers is prescribed to that device, like the service number or something like that. And like, so I could track it, and I could just track my participation just like a little survey card with two or three things to check off so I can make sure that I did all the things that I’m supposed to, and then I can send it off. I think that would be the only thing, maybe that I could add on. – CI-16

While most participants were comfortable with shipping blood samples, several concerns and logistical considerations remain. The success of home-based viral load testing would rest on successfully navigating mailing issues.

#### Considerations for sharing of test results.

We asked participants to describe how they would prefer to receive viral load test results. Most participants opted for the use of a secure web portal. Participants were split on the use of encrypted emails, phone calls, text messages and regular mail. Others mentioned the use of telemedicine which has increased in popularity with the COVID-19 pandemic. Two key considerations emerged: ensuring privacy and confidentiality of test results and providing options to participants for sharing of test results based on their preferences.

I believe in options and then it should be no, just one way to receive your results, but some people having the results put on to the patient portal or my chart might be a good go-to for me, that will work for me. For other people they might want to be told by the provider or the medical staff, where they go get out care or a phone call or during the visit from the provider. And they’re more comfortable hearing it from the medical team then going into a portal cause they might not know how to navigate it or even have it setup … We need to think about options more than just one way and making sure that people have like, they can get a phone call, they can get some people more savvy with the text messages. – FG-2, Participant #1

Most participants wanted their regular HIV care team to be made aware of viral load test results. Participants also recommended helping PWH navigate the meaning of their viral load test results.

Well clearly, in my opinion, whether I get an email from the lab, it’s got to be cc’d [copied] to my primary care physician because they’ve got to keep track of it, too. – CI-10

I’m talking about like my regular doctors that I see that treat me for HIV. I would want them to know everything about me, especially if they’re wondering why I’m not coming there anymore for my tests. – FG-2, Participant #2

One participant recommended including counseling and mental health support as part of sharing viral load test results.

I don’t know if you’re doing a link to care think where you’re making sure you have a therapist online, so on and so forth, as far as making sure that people with mental health is also staying well, because depression is also a big thing when it comes to HIV. So is foggy brain syndrome and a whole host of other things that, when things start to decline, people aren’t really thinking about, and it just starts happening naturally. So is that something that is also going to be integrated into this kind of program that you have for people that are doing this test study? – CI-09

There was no “one-size-fits-all” solution when sharing viral load test results with PWH. A tailored and patient/participant-centered approach will likely be necessary.

#### Considerations for technical support.

Participants provided suggestions to ensure optimal technical support for the home-based viral load test device. In addition to written instructions and a demonstration video, PWH recommended having a clear manufacturer website with frequently asked questions (FAQs). However, they also expressed concerns about both lack of computer literacy and access.

The whole COVID thing, my mother is not literate with computers at all, and there are people out there, believe it or not, that are not literate with computers. And I had to get her her COVID tests, because she couldn’t do it. And the only way to do it was through the internet … I mean, it’s fine to put it on a website, but I do think that there should be kind of some kind of phone system, whether it’s recorded or not with instruction, and an option to leave a message if they’re really that confused. – CI-02

Additional recommendations included setting up an automated or a live call center. Most participants preferred the live call option because it would be more personalized.

Just for myself, when it comes to my health, I need to talk to somebody. I don’t want to read nothing. I need somebody to talk to me. Well, in that case, I like the personal attention. I want somebody to talk to me in layman’s terms. Don’t be saying things that I don’t know. You barely know what they understand. I don’t want that. Don’t do that. Come down here where I’m at and talk to me. So, don’t be saying words that it takes up half the alphabet. So yeah, there should be a helpline. There has to be a helpline. – CI-05

I would say the difference between automated and having a live person to talk to is, automated is so impersonal. It’s like some text, when you get a text message, it’s so impersonal, it lacks empathy in me. I want something that I can sit and we can actually talk and I know who I’m talking to and we on the same page and we going through the same thing. And that gives me comfortability, that gives me assurance. – FG-1, Participant #3

Participants further recommended group sessions and live demonstrations, engaging personal HIV care teams and case managers, and using a combination of technical support options.

#### Additional suggestions and comments.

Participants provided additional suggestions and comments about the home-based viral load test device. Some wanted the device to appear less clinical, smaller, or more colorful. One participant suggested pairing the device with a health-related mobile app.

I think an improvement could be also, if this is hooked up to an app that you could have on your phone, so that every time you take this test, you can send a message to your physician, letting them know that you’ve taken it so they could expect in the mail your sample, but to have some kind of app that you could keep track of it for yourself on your phone.

– FG-2, Participant #5

Another participant recommended providing a subscription system for the device where it would be automatically delivered at home. Despite high acceptability of the device, some participants did not want to see phlebotomists out of a job and still perceived the need for in-clinic blood draws. Most participants were looking forward to trying the device.

## Discussion

Our qualitative study revealed high hypothetical community acceptability of a novel, home-based viral load test device in the context of HIV cure research involving ATIs in the United States. Findings have implications for successfully implementing home-based viral load testing in the context of ATI trials and regular HIV care. While HIV self-kits have long been available to detect the presence of HIV antibodies^15–20^ and home-based PrEP kits are now available,^31^ the HIV field is now exploring the development of a home-based viral load test device as a promising innovation and intervention. To our knowledge, this is one of the first studies to examine community acceptability of home-based viral load testing in the United States.

HIV cure studies with ATIs bring concerns around sudden viral load increases and the possibility of transmitting HIV to sexual partners.^10,13^ While PWH in our study were already conditioned to routine blood draws as part of regular HIV care, several reported aversions to needles or being inconvenienced by frequent clinic visits. The COVID-19 pandemic has precipitated hesitancy around frequent clinic visits for blood draws. Most participants in our study viewed the prospect for a home-based viral load test device as a timely, progressive, and significant. The device was perceived as a strategic next step to self-monitor one’s health and viral load at home. The home-based viral load test device would provide a sense of autonomy and self-efficacy, counter to some of the internalized HIV-related stigma. Participants made analogies with glucometers used by people with diabetes to regularly self-check blood sugars. Importantly, and unlike glucometers, the experimental home-based viral load test device would still require sending specimens to an outside laboratory. At this time, the device does not represent a self-test that could be conducted in one’s home with immediate results. Participants acknowledged the utility of the device would increase if it could provide an immediate point-of-care response, or if it would lead to additional measurements beyond viral load, such as CD4+ count, chemistries, cholesterol, and other STIs.

Perceived advantages of the home-based viral load test device included convenience, time savings, sense of control and independence, privacy, safety, anxiety reduction and no venipuncture (minimal invasiveness). Previous acceptability research examining home versus clinic-based specimen collections have similarly acknowledged the simplicity, security and privacy of self-specimen collection devices as important acceptability attributes.^18,23,25,26,30,40^ Additional facilitators to using the device included clear orientation materials that did not require high literacy (e.g., videos), thoughtfully designed features (e.g., easy grip), and reminder systems. These facilitators have likewise been noted in the context of interventions aimed at increasing STI self-testing.^23,27,41^ Additionally, and not included in our study, the ability to speak with individuals who have previously used self-sampling may help increase uptake.^27^ In the context of ATI trials specifically, home-based viral load self-testing could help obviate the need for frequent monitoring visits during ATIs, significantly reduce participant burden,^4^ and reduce anxiety associated with potential viremia. As described by Julg and colleagues, however, the precision in estimating time to viral rebound may depend on the testing frequency, which may in turn depend on the adherence of the ATI study participants to pre-agreed testing schedules.^2^ Further, the need for inclinic monitoring visits would also not be completely eliminated in the context of ATI trials.

Our study uncovered potential uptake barriers for the home-based viral load test device, such as concerns with physical side effects (visible marks on the arm), worries about not using the device correctly, blood aversion, logistical challenges (e.g., mailing, transportation), economic factors (e.g., insurance coverage), and increased isolation. Findings compare with prior research on attitudes towards self-testing that showed concerns around user errors or perceived lower accuracy of home tests.^18,25,29,42^ These apprehensions provide a window into potential anticipated challenges to successfully implementing this device in the context of ATI trials and regular HIV care. To facilitate uptake, previous scholars have recommended using simple and clear language, well-designed pictorial instructions, and intuitive self-test kits.^23^ An important acceptability factor will be sensitivity and fast availability of the viral load test result. While the ability to self-diagnose HIV detectable status – consistent with the highly popularized U = U campaign^40^ – was acceptable to some community members, most participants in our study desired the viral load to be as sensitive as possible, similar to commercial assays.

The main worry associated with HIV treatment interruptions was passing the virus to sexual partners while viremic.^10,13,43^ The availability of a reliable home-based viral load test would help mitigate these concerns. As suggested elsewhere, the device could be paired with counseling and support interventions to address psychosocial needs and concerns of participants during ATIs.^36^ Interestingly, as found in our study, the device may have mixed effects on stigma for PWH. While it may reduce stigma by providing increased sense of control, limiting the number of clinic visits and normalizing HIV as a chronic condition (like diabetes), the device also carries potential social risks, such as inadvertent HIV disclosure. A warning label about the possibility of disclosure was also recommended. From an acceptability standpoint, it will also be important to measure the effect of the device – as well as ATIs and viral rebound – on HIV stigma and quality of life for PWH.^36^

The logistical aspects of using the device may have a significant impact on acceptability. For example, potential considerations over mailing samples emerged prominently in our study, such as navigating same-day shipping, worries about misplacement or damage, timeliness, and de-identification. Private shipment companies were preferred by most participants due to their ability to reliably track packages. Proactively providing pre-stamping and pre-payment would also increase device acceptability. The feasibility and efficacy of sending dried blood spots by mail has been demonstrated in previous epidemiological studies.^44,45^ Successful implementation of home-based viral load testing would evidently be contingent on PWH being willing and able to send samples by mail. Moreover, an important theme was the provision of multiple options for study participants with regards to mailing samples, providing test results and technical support to match individual preferences. Maintaining privacy and confidentiality along the testing chain were also paramount considerations among PWH. Participants generously provided important suggestions to increase the future perceived value of the device, such as implementing assistance programs to understand the meaning of test results, providing concomitant mental health monitoring, support and care, telemedicine and health-related apps, and subscription systems. Importantly, participants noted the utility of the device may extend far beyond PWH involved in ATI trials. Several additional populations or groups were expected to benefit – including people with needle aversion, poor vein access, phlebotomy fatigue, disabilities, limited transportation, or living in rural areas.

**Figure 2.**
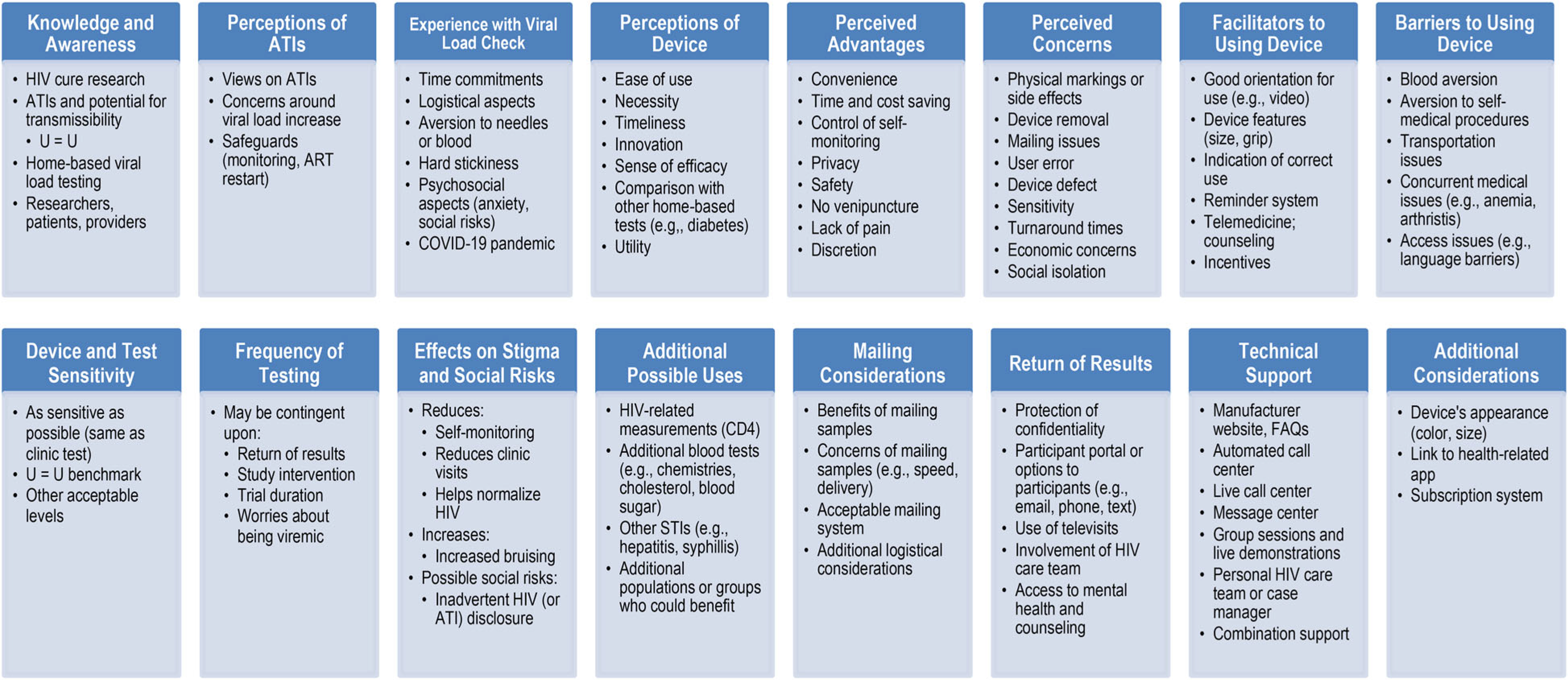
Preliminary conceptual framework for community acceptability of home-based viral load testing in the context of HIV cure-related research with ATIs.

Figure 2 provides a preliminary conceptual framework for community acceptability of home-based viral load testing in the context of HIV cure-related research with ATIs.

### Limitations

We must acknowledge limitations to our study. First, we assessed hypothetical acceptability of a home-based viral load test device. Results are based on perceptions rather than experiences. A separate study is underway to examine actual participant perspectives and experiences in the context of HIV cure trials involving ATIs in Philadelphia, PA, United States. Second, participants were self-selected, predominantly from the Philadelphia area and most had proximity to HIV cure-directed research. Therefore results may not be generalizable to the broader United States. Third, we conducted this study amidst the COVID-19 pandemic, which may have skewed responses for greater acceptability of home-based devices. Fourth, this study did not assess perspectives of biomedical HIV cure researchers or providers. However, a separate nationwide survey is assessing these viewpoints. Other limitations were inherent to the focus group methodology, such as potential for group think. Our study did not assess willingness to pay or acceptability of home-based viral load testing in resource-constrained settings. These areas are ripe for further research. These limitations notwithstanding, our study has several strengths, including the recruitment of a racially and ethnically diverse sample of PWH in the United States. After 15 community interviews and 3 virtual focus groups, we believed saturation was reached.^46^ We have also presented the findings with fidelity with respect to the rich qualitative data.

## Conclusions

The prospect of home-based viral load testing in the context of HIV cure research involving ATIs was strongly embraced by the community. If proven effective and reliable, the home-based viral load test device may become an important adjunct to ongoing HIV cure trials involving ATIs. Home-based viral load test will likely become a valuable intervention in the context of regular HIV care. As shown in our study, the pluri-potent uses of the device beyond viral load testing would present significant advantages. In the future, acceptability assessments will also need to be supplemented with implementation science frameworks and measurement tools to help evaluate the effectiveness of this device in real-word contexts. Pairing the device with telehealth options or mobile health interventions may also help optimize its impact. To be truly transformative, the device must help reduce disparities in access and outcomes in both research and care in all communities affected by HIV.

## Supplementary Material

Supplementary Table 1

## Figures and Tables

**Table 1.** Interview guide and focus group questioning route for Home-Based viral load acceptability study in the context of HIV Cure-Related research with ATIs (United States, 2021).

• Can you please tell us what you know about HIV cure clinical research? What do you think about interrupting HIV treatment?
• Can you discuss your experience going to the clinic and getting your viral load checked periodically?
• If proven effective, the home-based viral load test would allow people to measure their virus levels from home instead of going to the clinic. What would help you with getting your viral load checked? Do you think a home-based viral load test would be helpful?
• There is a device under development by a company called Tasso and Merck & Co, Inc., Kenilworth, NJ, USA. is sponsoring a study to understand acceptability of the home-based viral load test device that may eventually allow people living with HIV to test their viral load at home. This device is still being tested and is not yet available outside of research. [Show demonstration video] What are your thoughts about the video?
• What do you think of the experimental home-based viral load testing device? What were your first reactions when you learned about it? In your opinion, would this be helpful to participants in the context of HIV cure clinical trials involving interrupting your HIV medication? If so, how?
• Are you excited about this device?
• Do you have any concerns about this device?
• What would help participants in HIV cure clinical trials use the device at home?
• What would make it difficult for participants in HIV cure clinical trials to use the device at home?
• What are your thoughts about how good [or sensitive] you would like the test to be?
• How often should we ask participants to use the device at home in the context of an HIV treatment interruption?
• What are your thoughts about mailing in your samples? What are the benefits of mailing your samples? Do you have concerns over mailing your samples? Which method(s) would be most acceptable to you for mailing your samples?
• If this device gets approved and becomes available on the market, what would be the best way to share HIV viral load test results with people?
• Do you think there should be support offered to people who use the device? If so, what types of support should there be?
• Would you be interested in using this device? Would you be willing to use this device in the context of a clinical trial? Would you be willing to use this device outside of a clinical trial?
• Do you think there might be other uses for the device? If so, what are they?
• Do you think being able to test one’s viral load at home could help reduce stigma around HIV? If so, how?
• Do you have any final suggestions to improve the device?
• Do you have any other questions or comments about the device?

**Table 2a.** Demographic characteristics of interview participants (United States, 2021).

Participant Identification Number	Gender Identity/Sex Assigned at Birth	Age	Race/Ethnicity
CI-01	W/F	64	Black/African American
CI-02	M/M	57	White/Caucasian
CI-04	M/M	54	White/Caucasian
CI-05	W/F	61	Black/African American
CI-06	M/M	52	White/Caucasian
CI-07	M/M	61	Black/African American
CI-09	M/M	43	More Than One Race
CI-10	M/M	63	White/Caucasian
CI-11	M/M	58	White/Caucasian
CI-13	M/M	55	Black/African American
CI-14	M/M	33	Black/African American
CI-16	M/M	33	Black/African American
CI-17	M/M	43	Black/African American
CI-18	M/M	53	Other; Hispanic/Latino
CI-19	M/M	58	Black/African American

* W = Woman; F = Female; M = Man/Male.

* CI-03, CI-08, CI-12 and CI-15 did now show for interview.

**Table 2b.** Demographic characteristics of virtual focus group participants (United States, 2021).

Focus Group Number	Gender Identity/Sex Assigned at Birth	Age	Race/Ethnicity
FG-1	M/M	43	Black/African American
FG-1	W/F	59	Black/African American
FG-1	M/I	55	Black/African American
FG-1	M/M	61	Black/African American
FG-1	M/M	49	Hispanic/Latino
FG-2	TW/M	29	More Than One Race
FG-2	M/M	56	Black/African American
FG-2	W/F	50	White/Caucasian
FG-2	M/M	50	Black/African American
FG-2	W/F	59	Black/African American
FG-2	W/F	54	Black/African American
FG-2	W/F	57	Black/African American
FG-3	M/M	47	More Than One Race
FG-3	M/M	58	White/Caucasian
FG-3	M/M	61	More Than One Race
FG-3	M/M	41	More Than One Race
FG-3	M/M	62	Black/African American

* W = Woman; F = Female; M = Man/Male; I = Intersex; TW = Transgender Woman

## Data Availability

All data relevant to this study have been provided in the text and in the supplementary Appendices.
