# Supplementary material for: “We are looking at the future right now”: community acceptability of a home-based viral load test device in the context of HIV cure-related research with analytical treatment interruptions in the United States
Source: HIV Res Clin Pract. Author manuscript; Available in PMC 2022 Dec 22. (PMC9519804)
Supplement: Supplementary Table 1 [file NIHMS1796892-supplement-Supplementary_Table_1.docx]

**Supplementary Table 1: Home-Based Viral Load Acceptability Study in the Context of HIV Cure-Related Research with ATIs (United States, 2021)**

| **Themes and Sub-Themes** | | **Participant Number** | **Quotes** |  |
| --- | --- | --- | --- | --- |
| **Background Context** | | | | |
| **Perceptions of Analytical Treatment Interruptions** | | | |  |
| **Views around ATIs** | Favorable views | CI-02 | *I wouldn't have an issue with that. I've been on it for a long time and my results have always been good. So I'm not really worried about stopping. I don't think it's going to affect me. I don't know. I mean, I don't think that stopping HIV medications would be an issue if it was for a purpose in the end.* |  |
|  |  | CI-19 | *Yeah, I'd love to be able to do that, right? With some other type of a med that could keep my HIV under wraps. And so, that's like the only medication I take is the HIV pill. And so, if I can get rid of that one while not having to do a lot more, to keep the HIV under wraps, I would do it.* |  |
|  |  | FG-1, Participant #3 | *But would I stop? I've stopped for other reasons. So for me to stop it for clinical trials, for something different... Yeah I would. Because I already know the risk factors, of what can happen, in terms of resistance and all, so yeah. If I've stopped it for getting high, at least I can do something on a positive note, if it's going to create a cure, or for some information. Why not?* |  |
|  |  | FG-3, Participant #4 | *Yes. Nowadays, I think I would feel comfortable doing that, especially if I'm under the care of professionals, experts, scientists. I would, yes.* |  |
|  | Possible concerns around ATIs | CI-04 | *I know for a fact, by going off your meds and going back on it, the research shows, in the past, that is not good for the patient. That ends badly for the patient. It's not good, so if I had a more up and down situation ...* |  |
|  |  | CI-05 | *I'm skeptical of that. This is from me personally. I'm scared. I don't want to be not undetectable. I don't want to be detectable. I don't want that.* |  |
|  |  | CI-09 | *To be quite honest, as a person that's undetectable, it would probably be scary to me in the beginning, especially not having a certainty, not a guarantee. With my medications, it's tried, it's proven, I know it works. I've been very healthy when it comes to my HIV, so like I said, it would kind of throw me off a bit.* |  |
|  |  | CI-13 | *That's crazy. That's the most scary thing ever, are you kidding me?* |  |
|  |  | CI-16 | *That doesn't make me excited, and so, I know for me, I basically take what is considered a cocktail. And so there's more than one medicine in the pill that I take. But with that in mind, some of them last longer than others. And so, the expectation for me is to take it daily, so that none of them ever lapse in terms of how my body responds.* |  |
|  |  | FG-1, Participant #1 | *Well my thoughts about that. It's very iffy, and it's not advisable from my understanding for, you know what I'm saying, for the sake of my health to be taking a vacation, med vacations. Even though I am undetectable, but I still understand the severity of having to take my meds.* |  |
|  |  | FG-2, Participant #1 | *I'm going to be real transparent and honest. I have fears around it because my medicine has sustained my life and gave me a quality of life. And I know with treatment interruption, you have to come off your current medications in order to participate in the clinical trial... But I do have a lot of fears around stopping my medication to be in a clinical trial.* |  |
|  |  | FG-3, Participant #1 | *I think there needs to be carefully executed these types of analytical treatment interruptions, especially for those who may be taking other medications for other conditions. Because HIV medications also affect the drug levels of things like antidepressants, anti-anxiolytic and many other medications, so one needs to be concerned about how... what the impact that will have on someone when they actually stop those medications abruptly.* |  |
| **Need for ATI safeguards** | Close monitoring | CI-02 | *And if we knew that something was happening, for example, viral load started to change, then obviously we need to go back to some kind of method. Does that make sense?* |  |
|  |  | CI-05 | *But like I said, I would believe, I would hope that they're still taking blood, they're still making sure that you're okay, your numbers are good.* |  |
|  |  | CI-07 | *That's fine, because I'm being monitored. I don't have that added stress.* |  |
|  |  | CI-14 | *As long as I'm monitored, I think I can do it.* |  |
|  |  | CI-17 | *And I feel like, as long as it's being monitored closely, like viral load testing every week, then it shouldn't be a problem. Although, I wouldn't suggest to put somebody in the beginning. I'm talking about if you had it at least for 10 years or more, and you've been undetectable for the most part, that would be perfect for them. But not for someone that's just starting out.* |  |
|  |  | FG-2, Participant #4 | *Just briefly, I had to stop my medication when I ran out of options. So for three months I was not on medication. And for the issue of stopping the medication, I think that when you're on these trials, they are closely monitoring you. And the moment they realize that either stopping your medication or anything else you are doing might not be good for you, they're definitely going to pull you out. I think you are closely monitored by the doctors and everybody else.* |  |
|  | ART restart | CI-02 | *Obviously, if my viral load starts to increase, then I would want to decide what we're going to do.* |  |
|  |  | CI-05 | *And then, if there was a problem that they would put them right back on, because that's what I assume. But, I don't know because personally I've never done that.* |  |
|  |  | CI-14 | *So, I think I could deal with this because people are actually watching. And if I decide to back off and say, "No, I want my medicine again,"* |  |
|  |  | FG-2, Participant #2 | *Yes. My problem with stopping the analytical treatment interruption, when they would stop my medications, and I know that if something were to happen throughout your treatment while you're off your ART and you're just getting too low or whatnot, they can put you right back on them if that need be the case.* |  |
| **Involvement of HIV care provider** | | FG-2, Participant #1 | *I would need some more support around that. And that support would come from even my provider. Because I want my provider to be part of that. If I decide to go into the clinical trial, to be part of that process of monitoring what's going on with me. I want a collaboration between the researchers and my primary doctor, along with my medical team along the whole journey. So that way I'm being supported on every end as possible so that I can be real effective inside that clinical trial, if that's what I choose to do, but I do have fears around it.* |  |
| **Previous experiences with treatment interruptions** | Addiction | CI-02 | *A lot of years ago. I would say prior to 2010, I would be in and out of taking my medication, mostly due to addiction. But from 2010 on, I've been pretty consistent. I mean, I miss a dose here and there, I'm not going to lie, but I'm usually proactive about taking my meds.* |  |
|  |  | FG-1, Participant #2 | *Yeah, actually I did it for my active addiction, I did. Even though I seemed like the barber will actually take a while and the CD4 kind of stayed the same, but then was bouncing up and down. But then when I started taking my meds, I mean, the viral load went down, then the CD4 still stayed there and I've been watching it now.* |  |
|  | Travels | FG-2, Participant #5 | *I'd like to share a story. When I had reached an undetectable level for about a year, I had to take a trip and I forgot to bring my medicine on the trip. And so I didn't take it for a week until I got back home. When I got back home, I had slipped back into a detectable level and it took me almost nine months to get back to detectable, took me a long time. And so my fear is ... that's my fear of stopping, that it'll slip me back into a state that I don't want to be, and it'll take too long to get back. But if that issue could be addressed, I would probably feel more comfortable with it.* |  |
| **Experience with Viral Load Checking** | | | |  |
| **Conditioned for periodic blood draws** | | CI-06 | *Yeah, it's not a big deal. I've been doing it long enough… Before, it was more mental than it was physical. I never had a problem with blood draws and all that stuff, so it wasn't anything like that. It was just that reminder that you have to go and get it done in your head.* |  |
|  |  | CI-11 | *It hasn't really changed in terms of the procedure itself. But for me typically, just in terms of routine care, getting my viral load tested is part of my routine visits to my doctor.* |  |
|  |  | CI-14 | *And I go, well I still do go because I was programmed when I was not on medicine to go once every three months and I still do that once every three months. So, I'll go once a season.* |  |
| **Inconvenience** | Time commitments | CI-02 | *Annoying… It's a hassle. It is. First of all, it's time consuming, because you may have an appointment at nine o'clock, but you may not even get your blood until 10. They may not even get you in there until 10 o'clock. So, there's an hour spent. So, it's time-consuming*. |  |
|  | Logistical aspects | CI-11 | *For me, if anything, it's the logistics of having blood draw, would be the part that would be not uncomfortable, but inconvenient. So, if I have to do something or go someplace other than either the clinic or my doctor's office, then you have to factor that in. But the process of having blood drawn, for me, is not a big deal.* |  |
| **Needle aversion** | | CI-01 | *Well, I really don't like needles, but if it's for a cause I don't mind. I just take my little squeezy thing and squeeze it when they're sticking a needle in my arm.* |  |
|  |  | CI-04 | *For people that are afraid of needles, this device could be a lifesaver, because there are some people that would freak out over needles.* |  |
|  |  | CI-09 | *I hate getting my blood drawn. It sucks. It sucks. It sucks. I'm still a three-year-old in my mind. Every time I see the needle come out; I cringe. So that aspect for me I guess is something I'll never get over.* |  |
|  |  | FG-2, Participant #5 | *They usually for me get it the first time, but like a typical guy, I start crying as soon as I see the needle. And so I just ... I really tell them to try to distract me. So, they'll tell me, "Look this way," and they'll stick me and then it'll be over.* |  |
| **Hard stickiness** | | CI-18 | *I'm not afraid of needles, but the only thing I dislike is that, is very hard for them to find my veins, so they have to poke me several times, and it's just annoying and uncomfortable. But other than that, I don't have any other issues with that. It has always been difficult for the reason I just explained, yes. So, it's just pretty much just the same. But for some reason in the past few years, I have a feeling that it has gotten more complicated, for them to find the right place to poke me and draw blood. So sometimes they ended up doing it on my hand.* |  |
| **Social risks of being recognized as someone with HIV** | | CI-10 | *I mean back in the day, in the '90s, you were still trying to let no one know you were positive. Going to the phlebotomist was... First off there were clinics. Even back then there were doctors who specialized in HIV and you would see people waiting either at the doctor's office or at the phlebotomist's office and you kind of outed yourself because you could figure out why they were there.* |  |
| **Impact of COVID-19** | | CI-14 | *If the Corona or something else happens where we have to stay in doors, I prefer that than going out. So, I'd rather have had that this whole time than physically going, unless I'm sick, I did not want to go.* |  |
| **Perceptions of Home-Based Viral Load Test Device** | | | |  |
| **Perceptions of the Device** | | | |  |
| **Easy to use** | | CI-01 | *Looks easy and it doesn't take that long. Because it's easy to do and there's no real set up. And I just think it's easier for people to be able to test their blood. Because it's easier for them to get my blood samples and because you don't have to send it off and it takes a while sometimes to get it.* |  |
|  |  | CI-02 | *That first of all, it's easy to use, so there'd be no reason to go to a clinic to have it done… It's simple to use.* |  |
|  |  | CI-06 | *That first of all, it's easy to use, so there'd be no reason to go to a clinic to have it done… It's simple to use.* |  |
|  |  | CI-09 | *So yes, I would rather use this myself in my home than go to the doctors, to be quite honest. It's easy on the eyes. It's easier to use and it's more convenient. So yes, this is everything that a person would want in my situation when it comes to these kinds of things.* |  |
|  |  | CI-11 | *So I think that the acceptability would depend on whether it's as simple as what you see in the video. I think if it is, and you all you do is press the button and hold it for two seconds and that's it, that's pretty reasonable to expect people to do.* |  |
|  |  | CI-13 | *It's not complex at all. Seems like it's very simple and easy to use. It's user-friendly. That's the word.* |  |
|  |  | CI-16 | *I think just the simplicity of it, that it wasn't, again, a three-part process. It's almost as if you're putting on a Band-aid, to take it off. Or just it's not something to cover it up, you know what I mean? They're collecting a sample, so I like that it's a simple add-on, and just patiently wait. Yeah, that it's not a multiple step. So even putting the device together is not a thing, but after puncturing yourself, it's also not four more steps afterwards, it's very simple. I like cut and dry type things. It allows for transitions, easier, so I like that.* |  |
|  |  | CI-19 | *Pretty easy to put on, instructions were fairly easy. And so, it showed me how to do it, I think I could do it right now if I had one. You know, you put the things facing down, you pop it on your skin, you press it, wait for five minutes, peel it off…* |  |
|  |  | FG-1, Participant #3 | *Well, it was a win all the way around. I'm sorry. I didn't see a downside to that at all. Easy instructions. One, two, three method. It was simple, you can't go wrong. I find it practical.* |  |
| **Timely, innovative, progressive** | | CI-14 | *I thought, this is going to be something that would possibly be how medicine is going, how blood draws and stuff is going to go in the future…* |  |
|  |  | CI-16 | *I think it'll be progressive. I think it's a testament to the growth that has been done in research that's been done, and I think it will give, again, a lot of people hope because they'll see that progress is being made in the right directions. Where people don't have to be as dependent on hospitals and clinics and things of that sort.* |  |
|  |  | CI-17 | *It was a wow factor for me, because it's like, it's more advanced to do things like this at home, which is great. It's really great. But, I mean, I liked that the device is small. You know what I mean? It's a little discreet. The fact that you've got the security of doing it in the privacy of your home, all that's great.* |  |
|  |  | FG-1, Participant #3 | *My reaction was, it's about damn time. That was my reaction... . I find it very innovative… I find it really neat. It's a step in the right direction… We can all look at it where we once were and where we are with technology. And it's become really, really informative.* |  |
| **Sense of efficacy** | | CI-01 | *I can do it!* |  |
|  |  | FG-1, Participant #1 | *I would agree so. Like I said, I will be on board to utilize it for myself. I do believe that the outcome of having the device can help me monitor my viral load due to the fact that I'm not taking a regimen or anything. So I think will be helpful.* |  |
| **Similar to self-monitoring for diabetes** | | CI-18 | *Yeah, it made me think of people who do it like insulin, like the people with diabetes.* |  |
| **Device may not be for PLWH who enjoy interactions at clinic** | | CI-17 | *I'd rather go to the clinic. And that's just me, because I'm a little more personable. I need to talk and see people and things like that. You know what I mean? It's a little more the interaction thing. Especially with the whole COVID and being isolated from people now. And it's like, you need to go in there, you need to see people, you need to talk to people, you know? So, for that, I'd rather go to the clinic.* |  |
| **Useful during COVID-19 pandemic** | | CI-18 | *Big time. I'm actually an example of that because, I went for over a year without knowing what my viral load was, or my CD4 count was. And if I would have had that, I could have just do it at home.* |  |
|  |  | FG-2, Participant #1 | *Because when COVID hit, I had to go to the lab. I had some anxieties. And on top of the anxieties I already had about getting stuck. I had gloves on, double masks. I felt really ... My anxiety was on an level, I'm quite sure my blood pressure went up. So I think, yes, this would have been a perfect tool, tool box for people who have HIV, especially during COVID.* |  |
|  |  | FG-2, Participant #2 | *And unfortunately the first time I even stepped out to get my blood and see my doctor, I get a phone call the next Monday, "Oh, you've been exposed to COVID. You need to test and to quarantine." And well I only went to my doctor's and here to find out my doctor was the one who had it. While she was seeing me in a little office, we were this close to each other and he or she had COVID. So I wish this device was out at that time because of the anxiety after that situation happened, which was not very far into this pandemic. It was a couple months into the pandemic when nobody knew much about anything… This test would have been the perfect timing to come out during COVID.* |  |
| **Perceived Advantages of the Device** | | | |  |
| **Convenience** | | CI-02 | *I think that's a better method than going to a clinic, especially in cure trials, because you don't always have that ability to do that. This makes it much more convenient. Right. Now that would make this very convenient.* |  |
|  |  | CI-05 | *Because then they don't have to break up their schedule to go for doctor's visits. They can just do it at home. So, you put it in the mail, then boom is over. It would be much easier and much more convenient. That's my opinion. But, it would be much more convenient. Because, like I said, I work nine to five. So, if I have to take time off to go to a doctor to participate in a clinical trial, that's going to take time. I'm going to have to use my personal time. No. So now, all I got to do is hit, do it myself at home at my own convenience. I can even do it at my desk and put it in the mailbox. The post office is right around the corner, take it to the post office, drop it in the mail. That would be perfect. You can't get it any better than that.* |  |
|  |  | CI-07 | *But it seems really convenient, only takes five minutes.* |  |
|  |  | CI-09 | *When I first learned about it, I thought that that's something that's pretty great. Something that's going to be able to help me and I don't have to run all over the place… I also think about how convenient it would be for me as well. I wish I could do the trial studies for the device. To be quite honest, like I said, it's something that's convenient, super convenient, super easy, easy to hold. I have a face loofah thing that looks just like it. Just don't get it mixed up, I'll be fine using something like that. It fits in your hand. Like I said, even when it comes to the cartridge that fills up, once the cartridge is filled up, you send it and put it back. It's easy.* |  |
|  |  | CI-19 | *Well, me personally, if I needed to have blood drawn at the frequency of once a week, once every couple of weeks, maybe once a month, I think I probably would want to have this type of blood draw as opposed to getting in my car, going some place, waiting in a line, having my blood drawn… It's convenient. I don't have to leave my home to get blood drawn, and... I mean, not to get the blood drawn, I still have to drop it to the box to get it shipped. But, the actual blood draw can be done right here at this desk.* |  |
| **Time saving** | | CI-16 | *Probably time-wise. I'm somebody who's over-involved into 1001 things and so, if I could not have to schedule an appointment and then wait to be seen, and find parking. If I could do all those things at home, time-wise, I would. I would. It would seem more time efficient than anything else.* |  |
|  |  | FG-2, Participant #1 | *It seems really easy and simple and that's what I need in my life; really easy so I can keep it moving. Right? Because like I do multiple things in my community and I don't really have a lot of time. It'd be like rescheduling or trying to set up an appointment. And I think that I am capable of doing it because I basically already do something like this already. So I'm feeling good about this device.* |  |
|  |  | FG-2, Participant #4 | *Time-saving, it does save a lot of time. Sometimes I know when I was living out for, I would have to take two or three buses from King of Prussia to Paddington lockers.* |  |
| **Sense of control and independence of self-monitoring** | | CI-05 | *That I get to do it myself. I don't have to have somebody else draw my blood. I can do it myself. That's what I like the best. But that's one of the things that I liked the best. I like it that I don't have to go to the doctor's office. So now, I draw the blood myself, I send it into the lab.* |  |
|  |  | CI-16 | *So that, I liked the idea of independence.* |  |
|  |  | FG-1, Participant #3 | *It's become really, really easy as a tool to use to navigate your own health. You play a major part because you should, it's you.* |  |
|  |  | FG-2, Participant #2 | *This is something you can control. You do it when you want to do it, as long as it's sent out the time frame and everything else. So I think it's about having more self control about your own diagnosis.* |  |
| **Safety** | | FG-1, Participant #1 | *I think it will be safe enough to use.* |  |
| **No need for venipuncture** | | CI-09 | *I myself, because I like the fact that it's right there on your shoulder, I like the fact that it's not something searching for a vein.* |  |
|  |  | FG-01, Participant #2 | *One that caught my attention, there's not any needle needle going into a vein. And that does it, where they stuck it at, there's actually no vein.* |  |
|  |  | FG-3, Participant #4 | *I think for people who hate needles like myself, doing something like that is less intimidating and less scary. Even if I have someone else do it on me, like I will be willing. Sometimes I wonder how much I actually put off or do not even go to the doctor because I hate needles so much, and I wonder how many people actually feel that way. So I guess it will be less, like I said, intimidating, scary.* |  |
| **Did not appear painful** | | CI-05 | *But I don't think it should hurt that much.* |  |
|  |  | CI-07 | *It seemed to be painless.* |  |
|  |  | CI-16 | *It didn't look like it was painful.* |  |
|  |  | CI-18 | *I will feel less anxious, because I don't want to see a big needle, and if it's there I don't see it. I don't get to see the needle, I'm not afraid of needles, but I don't get to see it. So I don't how big the needle is, so it seems very simple and un-painful. I don't know if that's a word.* |  |
|  |  | FG-2, Participant #2 | *And I think someone mentioned about the pain and I actually seen this demonstrated on someone, they did it on themself to show us, and they said it was pretty much painless. I guess it depends on your definition of pain or your tolerance to pain, but that person expressed that it was very minor, simple. It's like a little prick, you would barely know, because it only stays on for a couple minutes. So I was told it's a very painless and invasive treatment to use. And I mean I would definitely use it if I had to.* |  |
| **Discretion** | | CI-07 | *You can do it at home with your own privacy* |  |
|  |  | CI-17 | *The fact that it's discreet, that you can do it at home and the size.* |  |
| **Perceived Concerns about the Device** | | | |  |
| **Concerns with bruising, physical marks or possible side effects** | | CI-01 | *It don't look like it hurts, it's just the bruising.* |  |
|  |  | CI-09 | *Even though the hole in the arm is a whopper. It is much bigger than a needle prick.* |  |
|  |  | CI-17 | *Just the pricking. And that's another thing about the video, it didn't tell you, what was the side effects of pricking?* |  |
| **Concerns with removing device from arm** | | CI-16 | *If taking it off, I would spew out blood, or just be leaking and not knowing what to do. I don't think that's what it is, but it seemed like it was simple enough. I couldn't see how deep it was, but just it looked pretty simple taking it off, but what if it's not that simple?* |  |
|  |  | CI-19 | *Well, not necessarily concern, but just seeing how she pulled the device off of her arm. It reminded me of a tick.* |  |
| **Delay with blood coming out** | | FG-3, Participant #2 | *So for me, that delay of not knowing if the blood is coming out.* |  |
| **Concern with using device incorrectly** | | CI-14 | *If you do something wrong or if you put it in an area that it shouldn't, yeah. That's my thing, if I did something wrong.* |  |
|  |  | CI-16 | *Maybe just the security of knowing that you did it correctly. Like, I trust that if I'm at a hospital or what have you, that they are trained physicians, that that's what they have gone to school for. Versus me just kind of like, "I'll try it." I don't like trial and error with health. I don't think that's fun. I don't want to just play Russian roulette with how well I'm doing. So, yeah I think that's the only apprehension, maybe. Like, there's some comfort in seeing somebody, knowing I did this correctly. Because like, if I was in a room with someone, I don't know that I could say, "Hey, did I do this, right?" Because I may not have time or the company whereas at a hospital, they're going to do it right, and I don't have to do it, so.* |  |
| **Quality or sensitivity of test results** | | FG-2, Participant #3 | *Well, no, because the professionals are there with the ... The physicians are there. Actually, when they take the vials, you can see when they go ahead and then they put it in the machine, they shake it up and everything, they label it. So I get to see all that stuff, all that process being done. And that makes me feel confident that I know that my bloodwork is going to come back at a good reading. So that way my physician will know everything that's going on with me and should be able to teach me and let me know exactly what's going on.* |  |
|  |  | FG-3, Participant #1 | *So my concern would be is how sensitive it would be? Will it be able to... Especially if you're doing it at home, it has to be sensitive or it has to be fool-proof. I don't know. Is this a sample that you get the blood and you send it in and it gets analyzed? If that's the case, then you've got the shipping and all the other conditions that may impact the results of the test. I would have to see that it actually compared equally to any of the other commercially available testing platforms.* |  |
| **Mailing issues** | | CI-09 | *Things get lost with FedEx and so on and so forth, delivered to the wrong address. But like I said, I really don't see any downsides to it other than human error… But that's about the only thing is the mail system.* |  |
|  |  | FG-2, Participant #1 | *Keep in mind, everyone ain't got money for stamps, right? So is the box going to be one of those like boxes where you don't have to pay postage?* |  |
| **Timeframes for receiving test results** | | CI-13 | *Or how soon will I know this? You know what I mean? Because at least if I go into a lab or go into the hospital or wherever, and get my blood drawn, I'll know at least by the next day or no more than two days, that I need to come back in to get blood drawn. In the mailing system, I won't know.* |  |
|  |  | FG-2, Participant #3 | *I guess I'll have anxiety of thinking about how's it going to get there? Is it going to get there in a timely basis? What's the results going to be like? And it won't give... There will be questions I will have.* |  |
|  |  | FG-2, Participant #5 | *I think what I meant though is, you were talking about you'd be worried about how it travels there to the place if you do it at home. And I'm saying, when you get it done at the lab or the clinic, do you concern yourself about where the viral piece goes in the hospital and how long it takes for the tests and all of those things?* |  |
| **Economic concerns (e.g., insurance)** | | FG-2, Participant #1 | *I'm always thinking about access, right? When we came out with the home test for testing for HIV, the first thing that popped in my head: access. What kind of access people going to have? Is this device going to be free of charge? Will it be something you can give with your insurance? And if you don't have any insurance, can you still get it through the pharmaceutical company that's making it or they're going to offer it that way because everyone is not going to be able to pay for this. So it should be made accessible to the community of people who have HIV. An issue that costs anything out of pocket. It needs to be something that they can get through the insurance; the insurance company need to know that they need to approve this because there's a need for this device.* |  |
| **Forgetfulness** | | CI-01 | *Sometimes I might forget and I don't want to forget.* |  |
| **Further social isolation (e.g., COVID-19)** | | FG-2, Participant #3 | *I think it's going to be another form of isolation. This is too easy. You just can just go online and just order something and just have it shipped and delivered. For me, I would think I would like to go out and be able to talk to the person that's sticking me or talk to the doctor and stuff like that. Let them go ahead and do that. So I think I prefer to have the service done.* |  |
| **Facilitators and Barriers to Using Device** | | | |  |
| **Facilitators** | Good orientation for use | CI-18 | *A good orientation on how to use it, or highlighting the advantages of doing it this way, or using this device… I guess, people feel less anxious in knowing that they can do that on their own, and get the same result.* |  |
|  | Device features – small size | CI-02 | *Maybe a little smaller… Maybe in the video, it just seemed like a very large thing to me, that maybe if it was a little smaller.* |  |
|  |  | CI-07 | *You could make it a little smaller, the device, if that's possible.* |  |
|  | Device feature – easy grid | FG-03, Participant #3 | *I'm guessing maybe making sure it's user friendly, meaning that it has a nice grip to it. Like hers is perfectly round, but maybe if they had like a place for your fingers, because people like me, I've had carpal tunnel surgery on both hands, and I also have arthritis.* |  |
|  | Indication that device used correctly | CI-06 | *You have to make sure everything's lined up and it's the right way. You're pressing it the right... Holding it long enough, those kind of things. If there was more indicator that you were actually... Not using it, I don't know, but that you've held it long enough, so that it's made that connection, if there's any way for that.* |  |
|  | Reminder system | CI-06 | *And then I would assume some reminder or system that says, "Okay, it's time for you to do your test today," and have a text message or something like that, that's tied into the whole program... The device itself seems to be pretty well designed.* |  |
|  | Use of incentives | CI-09 | *Some people might need incentive. Some people might need a trans pass to get mail. I know, in Philadelphia, a lot of people have a hard time getting around and that's why I was saying people who are a little bit more home bound, this is a really good thing to have. That might ... or some kind of voucher to help them do as far as the study is concerned.* |  |
|  |  | CI-14 | *Well, for the hesitant people, I guess, pay them. I think money incentive would work. Also not just money incentive, education, show them how it works at the office, use it on them there and have them do it there and say, "Look how easy it is." So, money incentives and have them do it in front and say, "See, you could do this at home.* |  |
| **Barriers** | Concurrent medical issues (e.g., anemia) | CI-09 | *I have what I need for me to get mail sent out and be brought in. I don't have to worry about some things that happen. I'm not anemic or anything else like that, so you wouldn't have to worry about loss of blood. Those are the extreme situations I could see for a person that was going through this trial that would need to be watched or looked after, but no, the product is super easy.* |  |
|  | Language barriers | FG-3, Participant #4 | *I'm thinking about my Latino folks who I think can actually benefit from this, is it going to be available in other languages or Spanish, in this case? Are we going to have a video in Spanish with people they can identify with, relate with too? And I think that will be something to consider because we want to reach out to other communities as well, like minorities.* |  |
| **Considerations for Viral Load Test Sensitivity** | | | |  |
| **As sensitive as possible** | | CI-05 | *It should be just as good as the clinic test. Definitely. Because I'm not going into the doctor's office so what I'm doing should equal if I went to the doctor's office. Yes, definitely.* |  |
|  |  | CI-06 | *I think it's got to be a sensitive as you can make it because you've got to be able to... You're looking for a way to stop to cure this disease so you've got to see where you are, especially if you're on this and you're not taking any medication. How's your body really... 200 is too high. I mean, I really think you've got to be able to take it down as low as you can and still have it be something that you can use.* |  |
|  |  | CI-11 | *But I know that lots of other people are used to a lower limited detection of 40 or even less and really stress out over blips and everything else in their viral load testing. I don't, but a lot of people do and so, I think in those cases, they probably would not be comfortable with a lower limited detection of 200. They would probably want to see something equivalent to what they receive when they go to the lab or the doctor's office or the clinic. So again, I think it comes down to where and why and how it's being used.* |  |
|  |  | CI-18 | *It should probably measure less than 200, otherwise people wouldn't trust it as much.* |  |
|  |  | CI-19 | *So, I think as sensitive as it can be in compared to the other tests.* |  |
|  |  | FG-3, Participant #4 | *Just as sensitive as whatever viral load test we have available, same.* |  |
| **Undetectable = Untransmittable (U = U) benchmark** | | CI-02 | *Like, I mean, I've seen viral loads that were really high… I would think within at least that 200 margin. Does that make sense? And then, obviously, you would be rating it based on the amount of the increase between the time periods.* |  |
|  |  | CI-09 | *I think it should follow the same standard as far as undetectable is concerned and untransmittable. As far as the rest is concerned, detecting copies and finding out exactly where your viral load and everything else like that is, I believe once you fall in that detectable range, if you're a person that's been healthy or if you're a person that's still going through it, maybe this is something that you need to consult with your doctor as far as making sure you're going back on your meds and things like that, for the simple fact that if you're taking this and you're right on with yourself, that's about the only thing that you really need to know.* |  |
|  |  | CI-10 | *This one, the results come back and it's U=U and 200 is the accepted parameter for U=U, I would be comfortable with that. I personally would be comfortable with that. I mean I would not insist. Again, I don't claim to be the physician to know whether it has to be 20, under 20 copies, under 50 copies. If our collected wisdom is below 200 is considered U=U, I'm comfortable with that, by myself.* |  |
|  |  | CI-16 | *I'm thinking of a few things. One of them being like, almost afraid to say that people like to play with fire. So if they know, "I'm at like, 198, 190, I can still," what have you, then they'll push the envelope. Versus the people who are trying to get below 200, so it might be a matter of perspective. Or maybe if there was like a way that there was a green light, yellow light type of an idea where it's like, "This is an average. You're kind of okay." And whatever helps you maintain that, but I don't know that, do viral loads change based off of activity or is it just meds intake? I think the U equals U level. I think that would be kind of sufficient, yeah.* |  |
|  |  | CI-17 | *I feel like the 200 mark is great. That's where it should be at. Because, like I said, I'm coming from 1998 where it was a thousand copies, then 200, then less than that. You know what I mean? So, 200 should be right where it is.* |  |
|  |  | FG-2, Participant #1 | *I think my mind instantly goes to U=U, undetectable equals untransmittable, right? … So I think a lot of education will be needed here… There needs to be some more conversation and community education around that piece. Right?* |  |
| **Other – higher levels** | | CI-07 | *Maybe about the 500 level… Well, I don't know what the consideration is now for the device and how sensitive it is, but I think it should detect the virus at a lower number than wait till it gets to 900 or something. I should think it should be more sensitive than that.* |  |
| **Frequency of Testing** | | | |  |
| **Once per week** | | CI-01 | *Maybe once a week or twice every two weeks or something like that. I mean if need be. To do this every day I don't think it's necessary because you're not giving it time to work. So maybe if you just separate the times a week to two weeks, then maybe you can get some results. I don't think every day should be done. That's too much sticking.* |  |
|  |  | CI-06 | *Well, like you said, I mean, if they're going to have to check it every week, I would say that once a week for three weeks, and then you go in for your followup, for your draw… I don't think that's too much to ask.* |  |
|  |  | CI-09 | *Well, I think once a week is pretty daggon good. Thank goodness it's mail in. Yeah, unless I'm going to get sick or something, once a week would be fine. I think once a week is a lot still. Every two weeks would probably be better, but once a week is great, especially if you're sitting there trying... this is a new product, you're sitting there testing. You want to make sure that it's working properly. So yeah, once a week sounds okay.* |  |
|  |  | FG-2, Participant #2 | *I thought it was one every week. At least ATI people that are on the ATI interruption, I thought it was once a week. So it's a fast process once you stop this medication. So I would say, that the only reason I say a week and not sooner, because you're not going to get an accurate read, as if you were a seven day stretch of no medication. Even if you get a slight variation of 10, you were like, it says 30, but now it's coming up 40. That detects some level that it's raised, even if it's a small, minor amount, it's still telling you something's happening within that week with the person not taking part and doing an interruptive therapy trial.* |  |
| **Bi-weekly (or every 15 days)** | | CI-16 | *I think it will be a matter of science, like how long does it take for the med to not be in your system? Because these are people who are interrupting their med, yeah, so I think at this point, for me, it would be like maybe a bi-weekly check-in just so I can monitor it and see how that's looking. I think a week wouldn't be enough time to really see any change in your body. You'd probably need at least seven days, just because some of the meds last longer than others. But I think the bi-weekly piece would be a nice check-in.* |  |
|  |  | CI-18 | *Probably at least every 15 days, I'd say. I think once a week will be probably too much. Every week for people, or for me. Every three days, every week, I think will be too much. Yeah, I think every two weeks, every 15 days will make people feel more comfortable.* |  |
| **Once per month** | | CI-17 | *I feel like a month to start with, and then it's... As long as everything's being monitored and it's not a significant change in percentage and things like that. You know the things that you need to look out for. If everything's still the same, then just lessen the months. You know what I mean? As long as everything's going great.* |  |
| **Every 6 weeks** | | CI-05 | *Because… you're on a trial. You've asked people to stop taking their medications. You need to be sure that they're okay… I think every six weeks is enough… So, if they have to do it, then definitely that machine.* |  |
| **Variables affecting frequency of testing** | Trial duration | CI-04 | *I think the longer the study, the more they should test the viral load. The shorter the study, the less they should test it. Again, it'd have to be an equation form. People that were in high risk, I think once a week… But I wouldn't make it go past two weeks, I think. Every week, I think, for the more in danger.* |  |
|  | Study intervention | CI-19 | *Well, it's going to depend on the study, so I would suspect in a study where you're not taking your medication, I'm not sure how often the HIV virus could change. You know, at least a week or two. Every week or two, you know, depending on how sensitive you want the data to come back. You may see a change after a week; I'm not scientist, but maybe a week or two.* |  |
|  | Worry around being viremic | CI-10 | *I know that you can't do it every day, but the biggest fear is once you stop your meds and start your ATI period, when's the switch going to throw? Two weeks is way too long. A week is much better, but a week, that would be predicated on how long it took me to get the results, because if I took a test every week but I don't get the results back for eight days, it's not every week, it's eight days, so it is a combination. I think that that is something that should be built into studies and to the informed consent of a discussion with the participant on what their comfort level is. People may want it more frequently.* |  |
|  |  | CI-10 | *This also ties into the whole conversations about sexual practices and unprotected sex that goes along with an ATI. These things are all interrelated. I just think that I feel strongly that there can't be or the ideal would be to not have just a one size fits all. You have to have a conversation with the participant and say, "How comfortable are you with us testing your blood every week when you're off your meds," and knowing that it's going to take X number of days to get your results, and have the ability built in to the study if somebody says, "I'm uncomfortable, can we do it every four days," that that's already been pre-approved so that you can do that to accommodate and alleviate the fears of the participant.* |  |
| **Possible Effects on Stigma and Social Risks** | | | |  |
| **Reduces stigma** | Reduces clinic visits | CI-01 | *Because a lot of times when you go to the doctors, people always want to know why you're going. And if it's not a clinic that does both, or if it's a clinic that does, then people are like stigmatizing you like, oh yeah, she got that thing or whatever.* |  |
|  |  | CI-02 | *I know in my situation, I'm involved with a AIDS care facility of some shape or form, but I think it would be great in those environments where people don't have that option, to use this for viral load testing communicated directly to their PCP [primary care provider] and then they don't have to be seen going in the door every three months for the lab work. Do you know what I mean?* |  |
|  |  | CI-06 | *But I think that in rural places, in places where it's not as just part of the normal course of business, course of your day, that people might feel more comfortable being able to do that at home and not feel like they're being stigmatized or feel like they have to walk in and ask for a test or something… Any way you can get that information out to people and get them treatment is important. Maybe they don't have the ability to get to a doctor or a clinic, or there isn't one. This at least gives them the opportunity to get some information back.* |  |
|  |  | CI-10 | *Stigma, now is it, can it reduce stigma? Sure, because I am, not me, but I believe that there are people out there who are still stigmatized or traumatized by having to go to the doctor and having to go to the phlebotomist, and doing some of these invasive tests at home, if that relieves that trauma or that stigma, that's a good thing. I mean I do understand, it's like going to the health clinic, I mean the public health clinic, where people feel stigmatized because either they don't have the money or whatever reason there is that they feel stigma for that, if this test reduces that by being able to do it in the comfort of your own home, that's a good thing.* |  |
|  |  | FG-3, Participant #3 | *I definitely think it would reduce stigma because the places you have to walk in to get your labs done sometimes are at the clinics that predominantly help people with HIV. And some people don't want to be seen there.* |  |
|  | Self-monitoring | CI-09 | *So it's the same thing with this device. When you have something like this that's able to put more control into your hands, and you're able to be responsible ... and that's the other thing. So once we sit there and we enlighten this, the products and the tools that are available, the knowledge that is being given to us, then that's when everything starts to subside when it comes to stigma because we'll start to be able to eradicate a lot of the ignorance that people have about these kinds of things.* |  |
|  | Normalize HIV as chronic condition | CI-16 | *It [HIV] might be normalized in a sense where it's not as discriminated against. It might be perceived as kind of people who are diabetic. Kind of like, it's just a thing you prick your finger and it is what it is. I'm being healthy, I'm checking my health and so on and so forth.* |  |
|  |  | CI-18 | *From my personal experience, it would allow me, because I'm comfortable talking about my HIV status, because I treat my HIV just like any other condition. So it would actually give me an opportunity to engage in conversations, and educate others. So if they ask me, I'll be like, "Yes, I'm just doing my viral load for HIV."* |  |
| **Possible social risks** | Limited social risks because device is discrete | CI-09 | *The device is very inconspicuous… As far as using it, like I said, I don't see how it could become an issue. I could see it becoming a talking point in your life where, if you have someone that is going to be significant with your or someone that you care about as far as family or friend, and you want to explain to them exactly how you're living and everything else like that, I think it's a really healthy and interesting way for a person to be able to explain what's going on with them.* |  |
|  |  | CI-16 | *I think it's about discretion, and so the same way I think people may or may not have their meds out when they have company, it would be the same place they keep their device… I don't think it would be an issue because it's not something that people are flaunting, and I don't think it's something that they're wearing as a badge of honor, necessarily that they are using the device.* |  |
|  | Inadvertent or forced disclosure | CI-01 | *Yeah, because people do... yeah, people do ask questions, you live with your family or whatever, and you haven't said anything to them. And then you go put this thing on your arm and they want to know why you doing it. And what do you say? You get stuck and nervous and really don't want to say anything about it. So just go to another room and don't ask me no questions and I tell you no lies. So again, it's the privacy thing.* |  |
|  |  | CI-05 | *Well, first of all, I mean, it wouldn't be left out in the open. It would be put in a private place and secured. Because that's a very important instrument. But, I can see some people if they don't have housing. But I would think they wouldn't give it to somebody that doesn't have housing because they would have no place to secure it. Because, like I said, I work in drug and alcohol. So, I know that some people don't have homes. Some people are renting a room for some... Some people have no privacy. So, I don't think it would be good for those people. I don't think so because they have a hard time securing their medications. So, securing that device would not be good.* |  |
|  |  | CI-10 | *Now keeping it in your home, how do we deal with that? I mean if you're living with somebody, either a roommate or a partner and you have not disclosed your status and you're afraid that getting a medical device will disclose your status.* |  |
|  |  | CI-10 | *That's why how it is delivered to you, it has to be, this is, you know we talked on that earlier. I mean at a minimum, your postal person is going to see that you're getting regular deliveries of medical devices, and that's a problem, but as long as the package is completely innocuous as to what the purpose of this is for, I mean we've done as much as we can on that front.* |  |
|  |  | CI-11 | *I could definitely see strange questions coming up, what is this? I could definitely see that. In the context of today, where this device is being used or considered for clinical trials, I would hope that anybody who's participating in a trial is comfortable with their status and being in a trial with new devices and new things. But if this were being used as part of routine care for people to monitor their HIV outside of a clinical trial, then yes, we have to think about all those different situations where not everybody will have disclosed to other people. They may not have disclosed their HIV status, they may not have disclosed their participation in a clinical trial, so it could create uncomfortable moments or a stigma or forced disclosure, it could do all of those things… Especially with long-acting injectable ARTs. So if people move in that direction of long-acting ART, so that they don't have a bottle of pills at home anymore, having this device might be cumbersome.* |  |
|  |  | FG-2, Participant #4 | *I have mixed feelings on this. If you live in the privacy of your own home, maybe it would be great, but if you live with other people, there's still stigma in some communities. There's still stigma in some families. So the concept would be, how would you create a safe enough environment without being discriminated against, or being kicked out by members of your family who did not know whether you're positive or not? Could you do it, is it portable enough that you would do it in the bathroom secretly without drawing the attention of everybody?* |  |
|  |  | FG-2, Participant #4 | *I don't know whether there's going to be a way around this. I don't know if this box is going to be, going to look different than other boxes, but like where I live, you just come out of the door and everybody's staring at you like that. So if you keep taking this box to the post office they're going to want to know. And I think there's going to be some stigma but I think I would suggest that maybe a lot of people going to that clinic or going for the blood.* |  |
| **Additional Potential Uses of the Device** | | | |  |
| **Additional HIV-related measurements** | CD4+ count | CI-04 | *I go to a center and I get all my blood work done at once. Why would I want a device if I'm only going to get my viral load? I need to know my T-cells too… It'd be great if you could do the viral load and the T-cells, that would be amazing, but that is a start. Hopefully down the road, they'll be able to accomplish the T-cells, because you really need to know them both.* |  |
|  |  | CI-07 | *It should be able to tell you what your CD4 count is.* |  |
|  |  | CI-14 | *Okay. Well, to me, because I would still have to, if I just want to do a viral load, I would still have to go in to test my CD4. So, that's what I'm saying. It would have to do that too. It needs to do both. I would. Yeah, because it would defeat the purpose, "Oh, that's your viral load, but we don't know what your CD4 is, so you have to come in and give us a bottle of blood for that."* |  |
|  |  | FG-3, Participant #4 | *I would like it to be used for CD4 count as well and viral load. That way I guess I don't have to go to the doctor from a... that often I guess. It'll be nice to if you could actually test for CD4 count as well.* |  |
|  | HIV resistance testing | CI-07 | *It should tell you what your viral load is, it should tell you what your CD4 count is, and if you're sensitive to any medication. Yeah. Those three things, they all go together.* |  |
| **Additional tests** | Chemistries | CI-11 | *I think that in the future, if it were possible to elect specimens for all the blood work that needs to be done, my routine chemistries and the CD4 count and whatever else is involved in either my routine visit to the doctor or the clinical study. If that were the case, that might make a big difference.* |  |
|  |  | CI-17 | *If it's taking blood, then you can test a lot with blood. You can test protein, you can test CBC [Chemistry Panel and Complete Blood Count], you can... The sky's the limit, I mean... You can get a lot of samples from blood.* |  |
|  | Cholesterol | CI-04 | *The cholesterol, the blood pressure, those are the four things as an AIDS patient. I think viral load, T-cell, cholesterol and blood pressure, those four you should know. At least twice a year, because those are just numbers you need… I'd like to see them expanded to the other ... The cholesterol, the lipid panel and everything else.* |  |
|  | Blood sugar levels | CI-07 | *It could measure blood for your blood sugar, kind of a dual thing.* |  |
|  |  | CI-09 | *Even when it comes to diabetes and stuff like that, but they have their own tracking system.* |  |
| **Other infectious diseases** | Sexual transmitted infections (e.g., hepatitis, syphilis) | CI-01 | *Maybe it can be a use for detecting any like venereal diseases or stuff like that. Sometimes people get these diseases and don't know that they have them and then it end up two, three weeks later, then they find out and sometimes it's too late. So maybe it would it be helpful for other things like that.* |  |
|  |  | CI-16 | *If there was an STI check or something like that. I don't know how much is collected in those capsules, but if there were one for I guess common STIs that people get, maybe. Yeah, I think that would be cool for people just to have, again, the cut-down time being at a clinic or hospital check-in. Yeah. I think just checking on their STI statuses.* |  |
|  |  | FG-3, Participant #4 | *The other thing that I wanted to add is I think that will be... it will be really nice to see it for syphilis as well… Sometimes people struggle to get an appointment to be tested for syphilis. And people freak out with HIV and syphilis, and it will be interesting to see that they can go to a pharmacy and just get a device to test it at home, which they have some, but I don't know.* |  |
| **Any type of blood tests** | | CI-05 | *Anything where your blood has to be tested. Any blood tests, really. Because it's giving your blood and you just send it and get it tested. So, any type of blood tests definitely.* |  |
|  |  | CI-10 | *Whatever disease or illness or whatever appropriate word that's not judgmental that requires blood draws, certainly this is there is this device can be used for that, also.* |  |
|  |  | FG-3, Participant #1 | *And the other major concern if, we're doing a cure trial, I would imagine that the investigators want more than just viral load. Typically, they want blood to do a variety of other tests. Is this just going to be an augmentation or are they just going to miss out on a whole bunch of other data points that we're telling them more about how we are responding to the treatments that we've just done?* |  |
| **Populations or groups that could benefit** | People with needle aversion | CI-05 | *I think, it would be beneficial for somebody, especially for people who are scared of needles. Because a lot of people are afraid of needles, so that would be better for them to get their blood drawn and not have to worry about it.* |  |
|  |  | CI-14 | *Or if some people are afraid of needles or afraid of somebody else doing it, they'll do it themselves because I know some people that have needle phobia and they'd rather do it versus let anyone else do it.* |  |
|  | People with phlebotomy fatigue | CI-14 | *I think if somebody gets what I call a sticking fatigue or phlebotomy fatigue, they would do it themselves.* |  |
|  | Older individuals or people with disability or mobility issues | CI-04 | *If I'm living outside the city and I can't get to them but once a year, because of my disability, because I have a wheelchair, because the clinic's not open, clinic burnt down or whatever, or I don't have a clinic, yes. Then at least knowing your viral load, that would help. So that does help the help.* |  |
|  |  | CI-09 | *I think it would work as far as a lot of people that are seniors or that have disabilities, that have a hard time getting around. I think this is a perfect program for them.* |  |
|  |  | CI-14 | *I think it would be good for senior citizens and older people to have or as I said, if you just can't get to the doctors and you haven't gotten a blood test in a while, it might be for emergencies or we have some variant of COVID comes back, we're in the house, that's what we would... it would be good for that.* |  |
|  | Homeless or unstably housed individuals | FG-2, Participant #4 | *How would somebody who's out there on the streets or whatever use it? Like homeless people, those who are not yet like stable mentally and all that. How would they use it? But I really think it's a brilliant idea. Yeah.* |  |
|  | People in rural areas | FG-3, Participant #1 | *For me, I live in a city. I'm close to my care providers. So it's no big deal for me to make a trip in to get blood drawn. But maybe for someone who is in a more rural, more remote area where it may be a 50 mile drive for their care, it might be advantageous in a setting like that.* |  |
| **Additional Logistical Considerations** | | | |  |
| **Considerations for Mailing in Samples** | | | |  |
| **Benefits of mailing samples** | No issue | CI-02 | *I don't have any problems with that. I've done that with stool samples, I've done that with several other methodologies. That's not an issue for me.* |  |
|  | Convenient | CI-05 | *The benefit that is convenient, it's easy.* |  |
| **Concerns of mailing samples** | Timeliness or speed of shipping | FG-2, Participant #1 | *I'm just concerned about the speed of it. Right?* |  |
|  | Lost in the mail or misplaced | CI-01 | *Getting lost in the mail, misplaced, crushed. Sometimes mail get opened and it's not really safe.* |  |
|  |  | CI-13 | *The most concern I would have is it being slow or losing it, but that's just the nature of that beast.* |  |
|  |  | CI-05 | *The concern would be that it got lost in the mail. Then you wouldn't have anything to judge that one particular time by it because you lost it in the mail. So then, they would have to do it again. Well, either wait until the next time around. So, that definitely is not a benefit if it got lost in the mail.* |  |
|  |  | FG-2, Participant #4 | *And one of the concerns would be, what if the package got lost and somebody got to know your status? That's a real challenge.* |  |
|  | Damaged or opened | CI-01 | *Like somebody took something out of it or the package broke and they have to re-pack it and sometimes things are missing from it. And I've had that a lot and I had to call FedEx or UPS and let them know that I don't know what happened to my package, but I know when I got it, it was open or some pieces was missing. And if they opened it by mistake there, sometimes they get thrown or dropped and they re-package it and it doesn't have everything in it, that it's supposed to.* |  |
|  |  | FG-1, Participant #2 | *But then what's going to happen if it gets out in the open? Like, this is going through the mail, the virus... What's going to happen if it gets out in the open and people come against it?* |  |
|  |  | FG-2, Participant #1 | *I think there might be some concerns around damaging packages. I've gotten those too, through the mail system, but I think the box that it's in special bag that is put in, the sample itself, which is a good thing.* |  |
|  | Extreme temperatures | CI-19 | *I don't know about how sensitive blood is to things like temperature. If you live in a colder area, will it affect the blood sample if you have to leave it in the mailbox? … I don't know if they're protected from the weather to not… Like, I was just in Las Vegas. 113 degrees, so if you put that in their mailbox, is it okay?* |  |
|  | Cultural beliefs | FG-2, Participant #3 | *I have two issues here in my community, like my African communities specifically, my community here in South Philly, we would not post blood… From those I've talked to, there's no way you're going to put your blood in the post office thing. Because there's a cultural belief of a bloody shoes.* |  |
| **Acceptable methods** | Private company | CI-06 | *That would be my choice. Prepaid label. You can drop it in one of their boxes. There's CVS, Walgreens, all of it, and you can drop it off anywhere really, any of the UPS, FedEx drop points.* |  |
|  |  | FG-2, Participant #5 | *I would agree with the FedEx or UPS.* |  |
|  | Private company – ability to track | CI-06 | *I think that would be okay because you would have a tracking number. You could still track it. If you're given the copy of the tracking number, you know you can go online and you can see where that package is even if you don't get a receipt.* |  |
|  |  | CI-09 | *But when it comes to FedEx, when it comes to private carrier, when it comes to things like that, I think you have a little bit more of a gage on it because it's easier tracked. So yeah, I like the fact that the mail can be tracked easier with FedEx, UPS, or with a delivery service.* |  |
|  |  | CI-14 | *A tracking number, a tracking way to track it.* |  |
|  |  | FG-2, Participant #2 | *And they give you a tracking number even, especially packages. So, I mean, I'm just saying to be safe than, sorry, I would probably want to pack a tracking number and everything.* |  |
|  | Private company – pick up times | CI-06 | *And the other thing is, is that with the other carriers, FedEx, UPS, you can drop it. Post office, the box is collected. You don't know when it's going to be collected. Is it going to get there? You never know. With these guys, you've got some way to know that it's really going to get there when it says it's going to get there.* |  |
|  | Dropbox – no dialogue | CI-10 | *I mean what you want to do is to make it so that you eliminate any personal dialogue. I just want to be able to drop it into a box. I do not want to have to go to the desk and have somebody sign off for it. Clearly, I don't know legalistically within each state, but the way it's got to be, you've got to try to eliminate any kind of person-to-person contact. Just dropping it into a box is what you want to strive for.* |  |
|  | Post office | CI-02 | *I would just drop it in a regular post office box. I would literally, as long as the package fit into the mailbox, the drop box, I would stick it right in the drop box.* |  |
|  | Post office – concerns | CI-04 | *I've officially stopped messing with the post office.* |  |
|  |  | CI-06 | *With the current way that US mail is handled? No.* |  |
|  |  | CI-09 | *I can honestly say that the way our mail goes in this country, that's an iffy subject to get ahold of, to really think about.* |  |
|  |  | FG-2, Participant #3 | *I will say concerns because the US postal service, they having issues with due to COVID. So how long will it take for it to get there? Will they ever get there for sure?* |  |
|  |  | FG-1, Participant #4 | *My concern is the postal service, how they is whack right now because of the pandemic and the different stuff with the political people shutting it down and stopping stuff. So, my concern is the frequency of the mail.* |  |
|  | Post office – long lines | CI-02 | *I don't mind going to the post office, but… you're in line forever.* |  |
|  | Home pick-ups | CI-06 | *I know it's more expensive, but if you can't get somewhere, have UPS come pick it up, or FedEx pick it up. I mean, they do offer pickups. If we're going to talk no cost here, then why not offer that as well?* |  |
| **Additional considerations** | Confidentiality  (de-identified) | CI-07 | *There's nothing on it that indicates for the public who you are. All there is a bunch of numbers and codes. So yeah, I think that would be pretty good. Long as it's done that way, I think it would be pretty good, as long as my name and that stuff don't show up on it.* |  |
|  |  | CI-09 | *As long as the samples come with a label, which I'm sure it already does, and not a name, and nothing personal of the person, it doesn't really matter.* |  |
|  | No mention of HIV | CI-10 | *One, is to make sure that the packaging that it comes in and goes out in is completely denuded of any kind of identifying marks with HIV.* |  |
|  | Pre-stamped or pre-paid | CI-02 | *And obviously, self-addressed and stamped… Postage. The postage should be on there so that you're just dropping it in the box. Everything's already done.* |  |
|  |  | CI-05 | *Well, I would hope that they are prepaid envelopes that you send them back… All you need is the post office to do their job. But if you got to peel off labels and all that kind of stuff, that's just too much. I might as well just go to the doctor. You're making me work too much.* |  |
|  |  | CI-06 | *I mean, the whole thing has got to be sticking... If Amazon has taught us anything, it's everything is done for you. Either you print it out or you even include it with the envelope and just slap it on and drop it. You make it as easy as possible to send the return in.* |  |
|  |  | CI-11 | *Having it all self-contained where all the materials to pack it up are there, instructions, and have it prepaid makes it simple so that none of those things become a barrier to returning the sample. I think that's what's probably most important.* |  |
|  |  | CI-16 | *Just I think the packaging, just insuring that the packaging is secure, and that the address is correct, that's it. I think that would be it. Just the formalities, the basic things.* |  |
|  |  | FG-1, Participant #4 | *Postage, return postage, something where I ain't got to spend money.* |  |
|  |  | FG-3, Participant #3 | *Prepaid postage. Yeah, whether it be FedEx, any delivery method, if they give you a label that's already paid for, you do it. You can call and pick it up, or you can drop it off at the closest location.* |  |
|  | Small packaging | CI-02 | *And it needs to be able to be put in the mailbox.* |  |
|  | Same-day mailing | CI-10 | *My only concern is will everyone have an easy way to get this to wherever the drop spot is, or can a home pickup be also part of it within the timeframe of it's got to be... I know that the chain has to start the same day. That would be my only concern.* |  |
|  | Mailing options | CI-11 | *I think it probably does make sense to have options because different people's situations may be different, so I think that having the ability to have it picked up from home or they could drop it off somewhere, whatever works better for them.* |  |
| **Considerations for Sharing Test Results** | | | |  |
| **Protection of privacy and confidentiality** | | | |  |
| **Portal** | Acceptable | CI-11 | *Again, I would think that having options would be a good thing because I'm comfortable using a patient portal and I would much rather do something on my computer than to fumble around with a tiny little screen on a phone. I have a smart phone, I know how to use it, I use it regularly, but I would much rather do something on my computer, that's for me. I know lots of other people who either don't routinely use a computer or simply prefer to do everything on their phone because they carry that with them all the time and that's just what they're used to doing. So I think either the option to receive a text message or to be able to check something from their phone is an option that a lot of people would like. But I personally would prefer to be able to look at a patient portal.* |  |
|  |  | CI-18 | *A portal will work, because I'm the only one who can have access to it, so I'll feel more comfortable accessing that information. It will be quicker, safest, confidential.* |  |
|  |  | FG-1, Participant #1 | *I'd rather them to have a result when they go to the visit or your portals are very secured and in the web. So accessing your portal if you've got my chart, you can go to my chart and look at the results of your tests yourself, that you're more comfortable doing that.* |  |
|  |  | FG-2, Participant #2 | *Well, they got that patient portal thing going on. I figure, what's wrong with that? I mean, something's already been all made. Why can't they have something like that?* |  |
|  |  | FG-3, Participant #4 | *A portal is probably a good idea… A portal I think is very convenient for me. That works really well.* |  |
| **Email** | Acceptable | CI-10 | *I personally love getting my stuff in email because you see I have telephone reception issues that suck, so for me, email is wonderful, but not everyone has email. Some people prefer talking to a person. I think you need a range of options that include at a minimum, telephone and email.* |  |
|  | Encrypted | CI-19 | *You know, I know that's probably sensitive information, so maybe an encrypted email.* |  |
|  | Not acceptable  (can get hacked) | CI-13 | *I mean, you can always email it to them, but I mean, I would say give them a choice. They could do both or whatever, so give them multiple choice because emails, nowadays, they're being hacked. And we all carry this, so we can instantly see whatever it is we need to see. You know what I mean?* |  |
| **Phone** | Acceptable | CI-14 | *I would say phone. I think that would be phone. Phone would be a good way. And as I said, it depends where it's being sent into, or if it's in conjunction with your medical doctor, then of course my doctor would, whatever they do.* |  |
|  |  | CI-18 | *To me personally, it could be over the phone, but I'm not sure that everybody else will feel comfortable in doing that. But it is something that we don't have available for other individuals.* |  |
|  | Not acceptable | CI-11 | *I really don't want a phone call.* |  |
| **Phone or texts** | Acceptable | CI-02 | *I'm fine with it either by phone or by text.* |  |
| **Phone or in person** | Acceptable | CI-17 | *I feel like it should be either on the phone or in person, not through mail or letter, because it's a little more personable than that. You never know what people are going to think once they get their viral load back.* |  |
| **Text messages** | Acceptable | CI-13 | *I think I probably would want to see like, a text or something. Yeah, that's me. A text and maybe my doctor.* |  |
|  |  | CI-19 | *I'm thinking a text would be good. I'm thinking a text would be good because then you could erase it, once you've got it, for privacy.* |  |
|  |  | FG-1, Participant #4 | *I would not mind getting my results in a text, but I would want my care team to give me a code either it's called blue or red or yellow colored rainbow. Or negative, I mean, funny textable or something.* |  |
| **Telemedicine or telehealth** | Acceptable | CI-07 | *I think a combination. You can do it with the person like you're doing with me, on Zoom, and you can have a printout sent to the person before you do it on Zoom, so they can look and ask you questions, and you can explain it. You see it in front of you, they see it in front of them. I think that's the best way to do it. That's what I do with my doctor.* |  |
|  |  | CI-09 | *I guess I would handle it with a Zoom call.* |  |
|  |  | CI-14 | *Televisits, I think it's called televisits. So, yeah, televisits, because that's what my doctor does after I get my results. I don't go all the way back down there for her to do it, She just... I read them, but then we go over them through a televisit.* |  |
| **Regular mail** | Acceptable | CI-09 | *The only reason why I say mail is because it arrives to your home. I've had some problem with technology being sketchy.* |  |
|  | Would be slow | CI-10 | *I mean snail mail, that's really slow and I don't think anybody, I would be surprised if people would say, "Send it to me in snail mail," so it's really going to be either phone. I mean am I missing something?* |  |
| **Providing options to participants** | | CI-06 | *Probably a text notification that your results are available and to tell you to link into either to some website or portal that you guys would have that's tracking all this information. And then I would think that you'd either have access to, or give access to your primary if they want to see it, or whoever. I mean, I think the texts via, and then with an email, with a link to the portal or an option to log in would be the best way to do it.* |  |
|  |  | CI-10 | *I think that again, that's something that needs to be discussed up front and there needs to be a variety of options. Not everyone has email, not everyone is comfortable getting their results via email.* |  |
|  |  | CI-19 | *But, you can also send them via their method of choice.* |  |
| **Involvement of HIV care team** | | CI-02 | *I think that the provider should be involved and should be aware of the result. Absolutely.* |  |
|  |  | CI-05 | *But, only thing I'm concerned about is, does my doctor get the information, too? Because they got to keep track and they got keep records of how I'm doing. It has to be documented.* |  |
|  |  | CI-11 | *I keep them involved in everything I'm doing anyway. I tell them about the clinical studies I'm in. I have the study, I'd share results with them, but that's just because I feel it's important to have everybody informed and aware of what's going on.* |  |
|  |  | CI-19 | *It would need to be a physician. Someone in the lab is just an associate. They're not a provider, and so, you know, I would have to work with the provider on that side in order to give me that information or have it sent to my provider and then have it sent that way.* |  |
| Help participants navigate meaning of test results | | CI-17 | *And then there's a lot of people you have to consider it, that's illiterate, that can't read, that don't know the instructions. So you need someone there to kind of coach you on through it. Or let you know, like, "This is what this means, but it doesn't mean..." Like, if you get your viral load back and it's 201, and it's like, "Oh my god!" You have to have someone near to be like, "That's okay." Because if you don't know, and you're just using the device... You have to be knowledgeable*. |  |
| **Considerations for Technical Support** | | | |  |
| **Manufacturer website or frequently asked questions (FAQs)** | Recommended | CI-05 | *Because some people are very, very curious. Some people come up with questions I would never think about. So, they need to have some way to go.* |  |
|  |  | CI-11 | *I think that an FAQ section on the website is a good idea. You may be able to answer some things up front and avoid having people remain confused or have to contact somebody with something that could be answered ahead of time. So, that's a good idea.* |  |
|  |  | CI-17 | *A FAQ would definitely be great… Give them a chance to read about it, click on it, get to ask some questions before, that would be great. I think that's a great way to do it.* |  |
|  | Not recommended | CI-10 | *For myself again, it cannot just be a website, but because not everyone has access to computers, and some people access computers by going to the public library where you can use a video. In my town, you can use, but nobody's going to want to look this up at the library because of even though you're in a carrel, there is no privacy.* |  |
| **Automated call center** | Recommended | CI-09 | *I think, at times, yes there definitely should be a call center. But when you initially start it, maybe send two the first time around just in case there is an issue with one accidentally breaking down on you, you always have a backup. Maybe that backup won’t work, but at least they have some kind of security, because a lot of people sometimes go into panic mode because we are dealing with people. Yes, a hotline with a 24-hour delivery, like Amazon Prime or something like that, would be awesome.* |  |
|  |  | FG-2, Participant #1 | *Yes, it should be a 1-800 number where people can call her in case they are struggling.* |  |
|  | Not recommended | FG-3, Participant #1 | *The automated systems usually are not easy and you don't often get your answer, and depending on your calling centers, they may or may not be helpful, or hopefully they would be. But you just never know.* |  |
| **Live call center** | Recommended | CI-06 | *Yeah, someone they could contact with the manufacturer that can speak to the issue in general. It's probably going to end up either user error or they'll have to send you out a new one.* |  |
|  |  | CI-11 | *I think an automated call center, probably not. I mean, if somebody's trying to draw blood and they're having trouble doing it, they don't want to talk to a machine or leave a message, they want to talk to a person. And my experience with chat bots is that most of the time, at least for the first several questions, you have to tell them things that you already explained in order to reach them in the first place, so that gets annoying. And in the end, they often cannot answer the question and have to transfer you to somebody else. So in this particular case, if we really want people to complete the process successfully and not abandon the process, I think they should be able to speak to a live person. Maybe at some point in the future, with more experience with the device, that won't be necessary. But I think at the beginning, it should probably be a live person.* |  |
|  |  | CI-16 | *Yes, it's just again, I like there's peace in knowing that someone can affirm that you're doing the right thing. And so, if there's like a call center or like a group just, I just would like a case manager or something like that, someone I could talk to directly to make sure that I'm crossing my T's and dotting my I's.* |  |
|  |  | CI-19 | *I wouldn't think a live call center would be needed, because it's just a device. A FAQ would probably be a good thing to have. Also, the video was very helpful to me… The video, the instructions, FAQ, I don't think it requires live support, but it may not hurt.* |  |
|  |  | FG-1, Participant #4 | *I think it should be a live call center, because it's so much better when you speaking to someone in person, because when things are automated, they are just automated and they're from a script. And then there may be questions that come up that the automation can't answer. And I think a live person would be better.* |  |
| **Group sessions or live demonstrations** | Recommended | CI-01 | *All of the above, and one more thing that I would add is a group session so that they can actually see and someone can actually demonstrate in person how to use it and see how effective it is.* |  |
| **Personal HIV care team or case manager** | Recommended | CI-10 | *I would be calling my doctor, who I have a relationship with and who I have a trust factor with, but I think that, again if you want to entice people to use this, yes, they should have somebody available if there are questions on how to properly use this to take those questions.* |  |
|  |  | CI-17 | *It can be a range from a nurse to a case manager. Just someone with more knowledge than the individual.* |  |
| **Combination of options** | Recommended | CI-10 | *It must be a combination… I guess you could have those websites where you can play the video and access a person who's going to walk you through the steps in the video. That's acceptable, along with having a call where you actually deal with a real person who can answer your questions.* |  |
| **Additional Suggestions and Comments** | | | |  |
| **Device’s appearance** | Make less clinical, different colors, smaller | CI-07 | *Pretty it up some. Don't make it look so clinical. What can I say? It looks really clinical. Give it some colors. Put some designs on it or something, so people say, "Ooh, it's a nice color." or even make it just a tad bit smaller. But pretty it up some, so it doesn't look so clinical.* |  |
|  | Smaller | CI-09 | *If you could make it a little smaller. But then again, that's just my take on my hands because I've seen it. It's a little bulky. It's not too bulky. It's fine. It's fine. Like I said earlier, there are people that have arthritis. It makes it easier for them to grip. So no, at this point in time, I wouldn't change anything about it. I like the red. Red is my favorite color.* |  |
| **Subscription system** | | FG-3, Participant #2 | *The other thing that I see as positive is that these days everything is... it has a subscription, for example, and you get a package every other month... It's in your home, confidential, nobody knows. And it's like a reminder. Every two weeks or every month you get like a razor subscription for something or... So it will be a very nice, marketable if you go that direction.* |  |
| **Do not put phlebotomists out of a job** | | CI-14 | *Don't put the phlebotomist of a job.* |  |
